# Triazole-Based Metal–Organic Frameworks for CO_2_ Capture

**DOI:** 10.3390/nano16150949

**Published:** 2026-08-01

**Authors:** Hafezeh Nabipour, Sohrab Rohani

**Affiliations:** Department of Chemical and Biochemical Engineering, University of Western Ontario, London, ON N6A 5B9, Canada; hnabipou@uwo.ca

**Keywords:** triazole-based metal–organic frameworks, CO_2_ capture, nitrogen-rich ligands, open metal sites, ultramicroporous materials

## Abstract

Metal–organic frameworks (MOFs) based on triazole have attracted considerable interest as promising porous materials for CO_2_ capture due to their high surface area, ultramicroporosity, and excellent thermal and chemical stability. Nitrogen-rich triazole ligands contain abundant Lewis basic sites that promote CO_2_ adsorption via dipole–quadrupole interactions, hydrogen bonding and cooperative interactions with open metal sites. The present review discusses recent developments in the synthesis of triazole-based MOFs, with special emphasis on the relation between structural features and CO_2_ adsorption performance. The paper reviews different synthetic routes such as solvothermal, hydrothermal, mechanochemical and post-synthetic modification methods and their impact on crystallinity, porosity and scalability. The roles of metal centres, pore confinement and linker functionalization in tuning CO_2_ uptake, selectivity and adsorption energetics are highlighted. Moreover, the mixed-linker strategies and defect engineering are explored to illustrate the use of the synergistic effect of nitrogen-rich sites and metal nodes for the improvement of the adsorption performance. Still, a number of challenges remain such as achieving an optimal balance between adsorption strength and regenerability, increasing stability in humid and realistic flue-gas conditions, and the development of scalable and sustainable synthesis routes. In summary, triazole-based MOFs provide a versatile platform for the design of high-performance CO_2_ adsorbents by combining structural robustness with chemically active, nitrogen-rich adsorption environments.

## 1. Introduction

Anthropogenic CO_2_ emissions remain the primary driver of global warming, with fossil fuel combustion representing the major source of these emissions. In 2024, coal-fired power plants accounted for 66.7% of global coal consumption and were responsible for approximately 41.6% of worldwide CO_2_ emissions. Consequently, the efficient capture of CO_2_ from flue gases has been identified as a critical first step in Carbon Capture, Utilization, and Storage (CCUS) technologies [1]. Among the various CO_2_ separation strategies, adsorption-based approaches have attracted considerable interest due to their relatively low energy consumption, operational simplicity, and potential for scalable implementation. Conventional porous materials, including activated carbons and zeolites, have been extensively employed for gas purification and CO_2_ capture owing to their low cost, commercial accessibility, and facile regeneration [2]. Nevertheless, these materials often suffer from limitations such as insufficient CO_2_ uptake under low partial pressure conditions, limited structural and chemical tunability, and, particularly for zeolites, pronounced moisture sensitivity that compromises CO_2_ selectivity and long-term cycling stability under humid operating conditions [2,3,4]. Alongside the development of conventional porous adsorbents, recent progress in structural and interfacial engineering has revealed the critical role of transport pathways, adsorption site distribution, and local chemical environments in determining the efficiency of separation processes. Emerging studies have shown that rational interface engineering, functional modulation, and confined transport strategies can effectively regulate molecular diffusion, improve separation selectivity, and reduce energy consumption in advanced separation systems [5]. These principles provide important design guidelines for developing metal–organic frameworks (MOFs) for CO_2_ capture, where adsorption behavior is governed not only by textural properties such as pore size and surface area, but also by the synergistic effects of chemical functionality, accessibility of adsorption sites, and confined molecular interactions within porous frameworks. Owing to their ultrahigh surface areas, large pore volumes, tailorable pore environments, open metal sites (OMSs), and high structural stability, MOFs have emerged as promising platforms for gas adsorption and separation applications. Furthermore, their well-defined crystalline structures enable systematic investigations of structure–property relationships, facilitating the rational design and optimization of advanced adsorbents [6].

The CO_2_ adsorption performance of MOFs is closely associated with their structural characteristics and chemical environments. Open metal sites (OMSs) act as strong Lewis acidic centers, promoting favorable interactions with CO_2_ molecules and enhancing adsorption affinity. Meanwhile, the introduction of polar or Lewis basic functional groups, such as –NH_2_ and –OH, into organic linkers can increase framework polarity and strengthen host–guest interactions. Tailoring pore dimensions and exploiting nanoconfinement effects further improve CO_2_ selectivity by promoting molecular sieving and enhancing CO_2_ confinement within restricted channels. In addition, defect engineering, particularly the generation of missing-linker defects, can increase the number of accessible adsorption sites and facilitate gas diffusion. The incorporation of multimetallic nodes provides another effective strategy for regulating adsorption strength through precise control over Lewis acidity and metal–CO_2_ interactions [7,8,9].

Linker chemistry is a fundamental aspect of MOF design, as modification of organic linkers provides an effective strategy to regulate framework topology, stability, and functionality by controlling structural geometry, connectivity, and the local chemical environment. Among the various linker families explored in MOF chemistry, triazole-based ligands have received significant attention due to their five-membered aromatic rings containing three nitrogen atoms and their versatile coordination behavior toward metal centers. Triazoles primarily exist in two tautomeric forms, namely 1,2,3-triazole and 1,2,4-triazole, both of which exhibit strong coordination capability with metal ions, including Zn^2+^, Cu^2+^, Co^2+^, and Mn^2+^, enabling the construction of robust and chemically stable MOF architectures [10].

Triazole-based MOFs have been investigated for a wide range of applications, including sensing [11], drug delivery [12], catalysis [13], dye adsorption [14], and gas storage and separation [15]. Their high nitrogen content and strong metal–ligand interactions provide abundant Lewis basic sites while contributing to enhanced framework stability and CO_2_ affinity. In particular, the electron-rich nitrogen atoms within triazole rings can promote favorable quadrupole–dipole interactions with CO_2_ molecules, which is advantageous for selective adsorption under practical operating conditions. Consistent with this concept, the incorporation of nitrogen-rich functional ligands has been reported to improve gas adsorption performance by increasing the availability of accessible basic adsorption sites [9,10].

The π-electron-rich nature of triazole ligands, arising from their delocalized 6π-electron aromatic system, provides both enhanced basicity and structural rigidity, thereby increasing the density of potential adsorption sites within MOFs [10,16,17,18]. Ping and co-workers reported a triazolato-carbonate MOF containing accessible nitrogen-rich triazole functionalities and exposed metal sites. The cooperative interactions between the Lewis basic triazole moieties and Lewis acidic Zn centers substantially improved CO_2_ affinity, leading to enhanced CO_2_/N_2_ selectivity. These findings highlight the potential of triazole-based MOFs as promising platforms for selective carbon capture applications [19].

Despite these advances, a comprehensive understanding of the relationship between triazole-based coordination environments and CO_2_ adsorption behavior remains incomplete. In particular, the combined effects of triazole functionality, metal ion identity, and pore architecture on adsorption capacity, selectivity, and thermodynamic characteristics have not yet been systematically established across different MOF families. While previous reviews have extensively discussed MOF-based CO_2_ capture from broader perspectives, including pore engineering and functional modification strategies, this review specifically focuses on triazole-based MOFs as a unique class of nitrogen-rich adsorbent materials. The central aim of this work is to establish detailed structure–property correlations linking triazole ligand chemistry, metal coordination environments, pore confinement effects, and CO_2_ adsorption performance.

In particular, this review examines how the synergistic contributions of triazole-derived nitrogen sites, OMSs, and ultramicroporous environments influence key adsorption parameters, including uptake capacity, selectivity, adsorption energetics, and regeneration behavior. Furthermore, the effects of different design approaches, such as linker functionalization, mixed-linker strategies, post-synthetic modification, and synthetic methodologies, are critically evaluated to identify the structural factors that determine CO_2_ capture performance under realistic operating conditions. Accordingly, this review provides a comprehensive assessment of triazole-based MOFs by correlating their structural characteristics and key textural parameters, including surface area, pore volume, and pore size distribution, with CO_2_ adsorption behavior. Particular emphasis is placed on understanding how triazole functionalization and metal center selection regulate adsorption mechanisms, including framework flexibility, host–guest interactions, and CO_2_ binding energetics. Through comparative analysis of diverse triazole-based frameworks, this work aims to establish general design principles for improving CO_2_ capture performance and guiding the development of next-generation nitrogen-rich MOF adsorbents.

Therefore, triazole-based MOFs provide a unique platform for understanding how interfacial chemical regulation, nitrogen-rich adsorption environments, and ultramicroporous confinement collectively govern CO_2_ adsorption thermodynamics, selectivity, and transport behavior; offering fundamental principles for the rational design of next-generation porous carbon capture materials.

## 2. Triazole Ligands and MOF Design in CO_2_ Capture

**Scheme 1:**
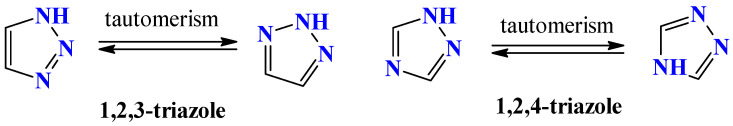
Tautomerism of 1,2,3- and 1,2,4-triazole.

Triazole-based ligands represent a versatile family of nitrogen-rich organic linkers that have been extensively employed in the construction of MOFs for gas storage and CO_2_ capture applications. Their aromatic stability, high nitrogen density, and flexible coordination characteristics facilitate the development of robust porous frameworks with diverse structural and functional properties. Triazoles predominantly exist in two isomeric forms, namely 1,2,3-triazole and 1,2,4-triazole, which differ in the relative arrangement of nitrogen atoms within the heterocyclic ring (Scheme 1). This structural difference leads to variations in electronic distribution, coordination behavior, framework topology, and ultimately the adsorption performance of the resulting MOF materials [20,21].

The chemistry of triazoles can be traced back to the late nineteenth century. The first reports of 1,2,3-triazoles were provided by Werner and Stiasny, while 1,2,4-triazoles were introduced by Bladin in 1885 [22]. Although these heterocyclic compounds were discovered at an early stage, their coordination chemistry remained relatively unexplored until the middle of the twentieth century. The first evidence of metal coordination involving 1,2,3-triazoles was reported in 1937, followed by the first crystallographically confirmed coordination complex in 1976 [23]. Since the beginning of the 2000s, triazole-based coordination chemistry has experienced rapid growth, particularly in the fields of coordination polymers and MOFs, owing to their versatile coordination capabilities and structural tunability [24].

**Scheme 2:**
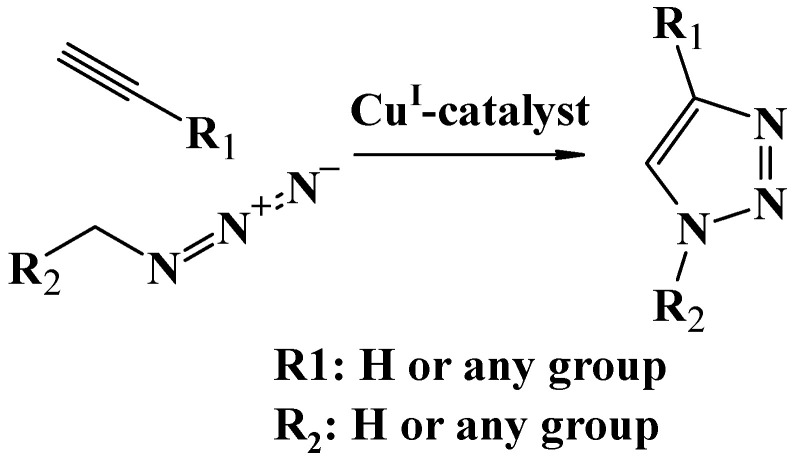
Copper(I)-catalyzed azide–alkyne cycloaddition for the synthesis of 1,2,3-triazoles.

**Scheme 3:**
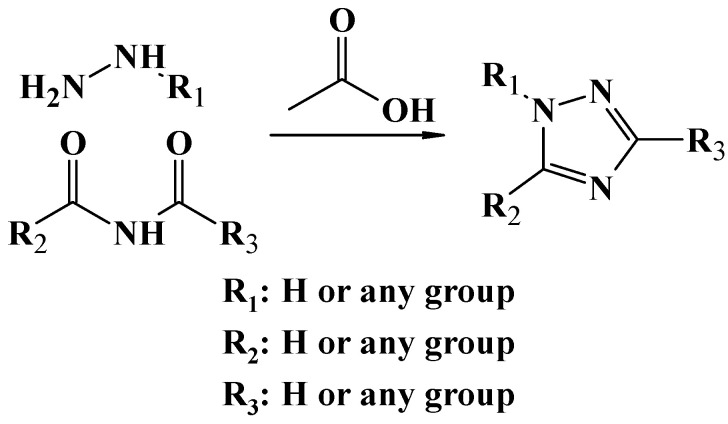
Einhorn–Brunner cyclization for the preparation of 1,2,4-triazoles.

From a synthetic viewpoint, 1,2,3-triazoles are predominantly obtained through the Huisgen 1,3-dipolar cycloaddition of azides and alkynes. The development of copper(I)-catalyzed azide–alkyne cycloaddition (CuAAC), commonly known as “click chemistry,” significantly expanded the application of this reaction by offering high regioselectivity, efficiency, and synthetic versatility (Scheme 2). In comparison, 1,2,4-triazoles are generally synthesized through traditional cyclization pathways, including the Einhorn–Brunner and Pellizzari reactions (Scheme 3) [25].

**Figure 1:**
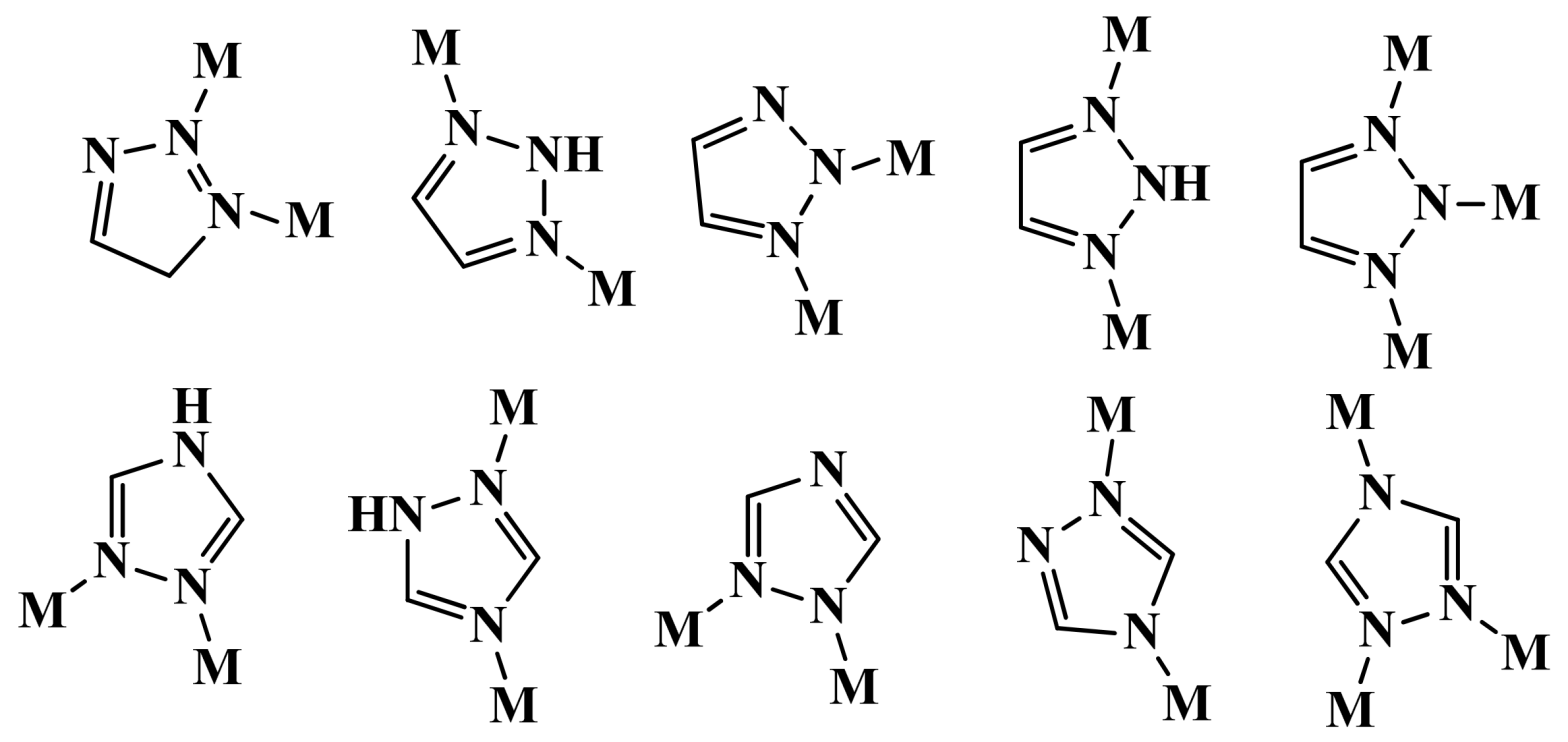
Various binding modes of 1,2,3-triazole and 1,2,4-triazole.

A key feature of triazole ligands is their exceptional coordination versatility. The nitrogen atoms within the aromatic ring provide multiple coordination sites for interaction with metal centers. Depending on the triazole isomer, protonation state, metal ion identity, and synthetic conditions, triazoles can coordinate as terminal ligands or act as bridging ligands to connect multiple metal centers [26]. As illustrated in Figure 1, both 1,2,3-triazole and 1,2,4-triazole derivatives display diverse coordination modes, facilitating the construction of coordination assemblies with various dimensionalities and topologies. In many cases, deprotonation of the triazole ring leads to the formation of triazolate species, which exhibit enhanced bridging ability and promote the connection of multiple metal nodes within extended frameworks. This coordination flexibility enables triazole ligands to generate a wide range of architectures, including one-dimensional (1D) chains, two-dimensional (2D) layered structures, and highly connected three-dimensional (3D) networks [10,27,28,29].

The versatile coordination behavior of triazole ligands with a wide range of metal ions further highlights their importance in MOF chemistry. Transition metal centers, including Zn^2+^, Cu^2+^, Co^2+^, Ni^2+^, Fe^2+^/Fe^3+^, and Mn^2+^, can readily coordinate with triazole-based ligands to generate structurally diverse framework architectures [30,31]. Moreover, triazole ligands can be incorporated into mixed-ligand systems in combination with carboxylate, pyridyl, phosphonate, and other donor functionalities. These hybrid coordination strategies provide additional opportunities for structural regulation and greatly expand the range of achievable MOF topologies. In particular, the combination of nitrogen-donor triazoles with oxygen-donor carboxylates enables precise modulation of framework connectivity, structural stability, and pore environments [10,27].

Another important advantage of triazole ligands is their facile chemical functionalization. The triazole ring can accommodate a variety of substituents while largely maintaining its coordination characteristics. Functional groups, including –COOH, –NH_2_, –OH, pyridyl units, alkyl chains, and aromatic moieties, can be incorporated to regulate steric effects, electronic characteristics, and ligand geometry. This modular functionalization strategy offers a versatile approach for the rational design of MOF structures. By tailoring linker length, rigidity, symmetry, and chemical functionality, the topology, pore dimensions, channel connectivity, and overall stability of the resulting frameworks can be systematically controlled [15,25,32,33].

The intrinsic rigidity of triazole-based ligands is another critical factor influencing framework formation. In contrast to flexible organic linkers, aromatic triazole units provide rigid coordination motifs that promote the generation of well-defined crystalline frameworks. This structural rigidity improves the predictability of framework assembly and contributes to enhanced thermal and chemical stability. Furthermore, the aromatic nature of triazole rings enables additional non-covalent interactions, including π–π stacking and hydrogen bonding, which can further reinforce framework integrity and affect crystal packing arrangements [33,34].

Triazole ligands enable the formation of a broad spectrum of MOF topologies due to their diverse coordination modes and adaptable geometries. Depending on ligand configuration and the coordination preferences of metal centers, triazole-based MOFs can exhibit various structural motifs, including pillared-layer frameworks, zeolite-like architectures, cubic networks, interpenetrated structures, and highly connected three-dimensional frameworks [35,36,37]. In pillared-layer systems, triazole ligands function as bridging spacers that connect two-dimensional metal–organic layers to generate three-dimensional porous structures. Zeolite-like triazole frameworks typically possess well-defined channels and enhanced structural stability, whereas more complex architectures may display structural flexibility or breathing behavior in response to external stimuli. This structural versatility demonstrates the significant potential of triazole ligands for advanced framework engineering [24,25].

To further extend the structural diversity of triazole-based MOFs, numerous triazole-derived ligands have been developed through rational ligand design. Representative examples, including 4-(1,2,3-triazol-4-yl)-benzoate [38] and 3,5-bis(isophthalic acid)-1H-1,2,4-triazole [39], illustrate how subtle modifications in ligand structure can markedly influence framework connectivity, porosity, and CO_2_ adsorption properties.

In summary, triazole ligands serve as versatile building blocks for MOF construction, owing to their rich coordination chemistry, synthetic accessibility, structural rigidity, and nitrogen-rich characteristics. These features enable the rational regulation of framework architecture and functionality. Importantly, the electron-rich nitrogen atoms within triazole rings can establish favorable interactions with CO_2_ molecules through quadrupole–dipole and Lewis acid–base interactions, thereby enhancing CO_2_ affinity. Collectively, these structural and chemical advantages make triazole-based ligands highly attractive for the development of porous frameworks with tunable adsorption performance.

## 3. Synthetic Methodologies for Triazole-Based MOFs and Their Structural Characteristics

A variety of synthetic strategies have been developed for the preparation of triazole-based MOFs, with each approach offering specific advantages in terms of crystallinity, morphological control, reaction efficiency, scalability, and environmental sustainability. Among these methods, solvothermal/hydrothermal synthesis, microwave-assisted synthesis, and mechanochemical approaches are the most commonly utilized. The selected synthetic pathway plays a crucial role in governing framework formation, influencing key structural features such as crystallinity, particle size, porosity, and ultimately the CO_2_ adsorption performance of the resulting materials.

### 3.1. Solvothermal and Hydrothermal Synthesis

Solvothermal synthesis is one of the most widely adopted strategies for constructing triazole-based MOFs, owing to its versatility and ability to generate highly crystalline frameworks with well-defined structures. In this approach, metal precursors such as Zn^2+^, Cu^2+^, Co^2+^, Ni^2+^, and Cd^2+^ are combined with triazole-containing organic ligands in high-boiling-point solvents, including dimethylformamide (DMF), dimethylacetamide (DMAc), diethylformamide (DEF), or alcohol-based solvents. The reactions are typically conducted under sealed conditions at temperatures ranging from 80 to 200 °C. The elevated temperature and autogenous pressure promote controlled nucleation and crystal growth, facilitating the formation of coordination frameworks with high structural order [40].

For instance, a solvothermal reaction between Co(NO_3_)_2_·6H_2_O and 1,2,3-benzotriazole-5-carboxylic acid (H_2_L19) in an acetonitrile/water solvent system at 150 °C resulted in the formation of the hydrated framework [Co_3_(L19)_2_(OH)_2_]·3.7H_2_O [41]. In this structure, the carboxylate groups of the ligand function as dinucleating bridges, whereas the triazolate moieties coordinate with three Co(II) centers through a μ1,2,3 coordination mode. Additional hydroxide bridges connect cobalt centers to form Co_3_(OH)_2_ chains. The subsequent connection of these chains by L19^−^ ligands leads to the formation of a neutral three-dimensional network containing one-dimensional rhombic channels oriented along the crystallographic *c*-axis [41].

Hydrothermal synthesis, in which water is employed as the reaction medium, represents an alternative approach particularly suitable for ligands with limited solubility in organic solvents. For example, hydrothermal treatment of 1,2,4-triazole (HL21) with CuSO_4_·5H_2_O in water at 200 °C for 48 h afforded [Cu_3_(μ_3_-OH)(L21)_3_(OH)_2_(H_2_O)_4_]_n_·4.5n(H_2_O) [42]. The resulting structure comprises trinuclear Cu(II) clusters that serve as secondary building units and assemble into a porous three-dimensional framework with accessible channels and potential host–guest interactions. Furthermore, this framework exhibits notable magnetic properties arising from intra- and intercluster antiferromagnetic interactions [42].

Therefore, solvothermal and hydrothermal methods remain highly effective for producing crystalline triazole-based MOFs with reproducible structures. Nevertheless, their relatively long reaction times, elevated energy consumption, and reliance on organic solvents may impose limitations for large-scale and sustainable production.

### 3.2. Microwave-Assisted Synthesis

In addition to conventional solvothermal routes, alternative energy-efficient methods have been developed to improve reaction kinetics and reduce synthesis time. Microwave-assisted synthesis has emerged as a promising alternative to conventional thermal methods owing to its ability to substantially reduce reaction times and improve energy efficiency. Unlike traditional heating, microwave irradiation enables direct and homogeneous heating throughout the reaction medium, thereby accelerating nucleation and crystal growth processes [43]. Consequently, synthesis durations that conventionally require several hours or days can often be shortened to only a few minutes. A representative example involves the preparation of a cobalt-triazole framework, [Co_2_(triazole)_3_Cl], through microwave-assisted synthesis using CoCl_2_·6H_2_O and 1,2,4-triazole dissolved in deionized water [44]. Structural analysis indicated the presence of two distinct cobalt coordination environments, designated Co1 and Co2. Co1 exhibited octahedral geometry through coordination with six triazole nitrogen atoms, whereas Co2 adopted tetrahedral geometry through interaction with three triazole nitrogen atoms and one chloride ligand. Under alkaline conditions during oxygen evolution reaction (OER) testing, chloride exchange with hydroxide species occurred at the Co2 site, potentially generating catalytically active centers responsible for enhanced oxygen evolution performance [44].

Compared with solvothermal methods, microwave-assisted synthesis often gives smaller particles and much shorter crystallization times. However, phase purity and crystallinity can be more sensitive to reaction parameters, and scale-up remains challenging.

### 3.3. Mechanochemical Synthesis

Mechanochemical synthesis has recently attracted considerable attention as an environmentally benign and industrially attractive strategy for MOF fabrication. In contrast to solvent-intensive techniques, this approach relies primarily on mechanical energy to induce reactions between metal sources and organic ligands, frequently in the presence of small quantities of liquid additives through liquid-assisted grinding (LAG) procedures [45]. Subsequent aging or mild thermal treatment may be employed to complete framework development. For instance, Zn-TRF was prepared using a low-solvent mechanochemical route involving liquid-assisted grinding followed by methanol incubation [46]. In this synthesis, basic zinc carbonate was mechanically processed together with fumaric acid and 1H-1,2,4-triazole, leading to the generation of a highly porous coordination framework. Structurally, the material consists of binuclear Zn^2+^ centers exhibiting distorted tetrahedral coordination geometry. The triazolate ligands bridge neighboring metal centers to create two-dimensional coordination sheets, while fumarate molecules act as pillar linkers connecting adjacent layers and extending the assembly into a three-dimensional architecture. This arrangement produces interconnected microporous channels with multiple adsorption environments determined by framework topology and linker orientation [46]. Mechanochemical methods offer clear advantages, including low solvent consumption, reduced energy input, and high potential for scalability. Nevertheless, challenges such as phase purity control, limited structural predictability, and difficulty in accessing complex topologies still restrict broader applicability. Despite its green and scalable nature, mechanochemical synthesis often leads to challenges in structural predictability and crystallographic control.

Table 1 presents a comparative summary of the major synthetic approaches employed for the preparation of triazole-based MOFs, emphasizing their respective effects on structural control, reaction efficiency, environmental sustainability, and scalability. As highlighted in Table 1, each synthetic route offers specific advantages while also presenting inherent limitations; therefore, the selection of an appropriate methodology requires careful consideration of crystallinity, productivity, sustainability, and practical applicability. From the perspective of large-scale production, no single synthetic strategy currently meets all the requirements for the industrial manufacturing of triazole-based MOFs. Solvothermal and hydrothermal methods remain the most established approaches due to their excellent control over framework formation and high reproducibility. However, their reliance on batch processes, organic solvents, and elevated energy consumption can restrict their scalability. Microwave-assisted synthesis provides notable improvements in reaction efficiency and space–time yield, particularly when integrated with continuous-flow systems, although equipment costs and challenges associated with reactor scale-up remain significant obstacles. Mechanochemical synthesis represents a promising alternative owing to its minimal solvent requirements, reduced energy consumption, and compatibility with continuous manufacturing processes. Nevertheless, further improvements in phase control, activation reproducibility, and process standardization are necessary before these approaches can be broadly adopted for industrial-scale MOF production.

In summary, the choice of synthetic methodology plays a decisive role in defining the structural and functional characteristics of triazole-based MOFs, including crystallinity, particle morphology, and pore architecture. These properties directly affect CO_2_ adsorption behavior by regulating accessible surface sites, molecular diffusion pathways, and host–guest interactions. Consequently, rational selection and optimization of synthesis strategies are essential for the development of high-performance MOF adsorbents for efficient gas capture applications.

## 4. Physicochemical Properties of Triazole-Based MOFs for CO_2_ Capture

Triazole-based MOFs have emerged as a promising class of porous materials for CO_2_ capture owing to their unique combination of structural and physicochemical characteristics. These features include high thermal and chemical stability, tunable porosity with large accessible surface areas, facile chemical functionalization, and the ability to incorporate specific active sites for catalytic CO_2_ conversion. The integration of triazole ligands, including 1,2,3-triazole and 1,2,4-triazole, into MOFs introduces nitrogen-rich chemical environments that promote strong interactions with CO_2_ molecules through dipole–quadrupole interactions, hydrogen bonding, and coordination with OMSs. These synergistic interactions play a key role in enhancing CO_2_ adsorption capacity and selectivity.

### 4.1. Stability

**Figure 2:**
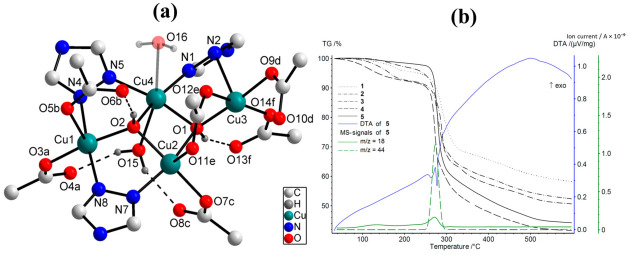
(**a**) Structural illustration; (**b**) TG-DTA-MS analyses of copper-based triazolyl isophthalate MOFs. Reprinted from Ref. [49].

Stability is one of the most crucial factors determining the applicability of MOFs in CO_2_ capture processes, especially under real industrial conditions such as flue gas, natural gas purification, and biogas upgrading [47]. Exceptional thermal and chemical stability of triazole-based MOFs is largely attributed to the strong coordination bonds formed between the triazole ligands and metal ions. High thermal stability, at temperatures exceeding 300 °C is a result of the rigid, nitrogen-rich triazole rings that enhances the strength of the metal-ligand bonds [48]. The Cu-based triazolyl isophthalate MOFs, with the general formula ^3^_∞_[Cu_4_(μ_3_-OH)_2_(R_1_-R_2_-trz-ia)_3_(H_2_O)_x_], are synthesized via solvothermal or reflux methods using Cu(II) salts and functionalized triazole linkers (Figure 2a). Their structures are constructed from [Cu_4_(μ_3_-OH)_2_] secondary building units, forming 3D microporous frameworks where pore size and accessibility are tuned by alkyl substituents on the triazole rings. Thermal stability was evaluated using temperature-dependent powder X-ray diffraction (TD-PXRD) and thermal analysis (TG-DTA-MS). As shown in Figure 2b, the frameworks remain structurally stable up to ~180–230 °C, with decomposition temperatures increasing to ~250 °C for bulkier substituents. Initial weight loss corresponds to solvent removal, while higher temperatures lead to ligand decomposition. Enhanced thermal stability is attributed to steric shielding and increased rigidity induced by linker substitution. This stability is crucial for CO_2_ adsorption, as it enables successful thermal activation under vacuum without framework collapse, thereby preserving porosity and ensuring reliable gas uptake performance [49].

Triazole ligands impart exceptional resistance toward both acidic and basic environments, enabling triazole-based MOFs to retain their structural integrity under the highly corrosive conditions often encountered in industrial CO_2_ capture processes. This characteristic is particularly advantageous for post-combustion CO_2_ capture from flue gases, where the presence of acidic impurities such as SO_x_ and NO_x_ can accelerate the degradation of conventional MOF structures [50]. A representative example is FJI-H14, synthesized from 2,5-di(1H-1,2,4-triazol-1-yl)terephthalic acid (H_2_BTTA) and Cu(NO_3_)_2_. This Cu(II)-based MOF exhibits remarkable thermal and chemical stability, which is primarily attributed to its square-pyramidal Cu(II) coordination environment involving Cu–N (triazole) and Cu–O bonds. In contrast to conventional Cu–O paddlewheel-based structures, the stronger Cu–N coordination interactions provide enhanced resistance against hydrolysis and thermal degradation. FJI-H14 preserves its structural integrity in aqueous media across a broad pH range (2–12) at 373 K for 24 h, owing to the formation of CuN_2_O_3_ coordination units and the stabilizing effect of uncoordinated nitrogen sites. This combination of structural robustness and chemical stability contributes to its excellent recyclability and CO_2_ adsorption performance [51]. Similarly, Lu et al. demonstrated that MAF-X27-Cl (Co_2_(μ-Cl)_2_(bbta), H_2_bbta = 1H,5H-benzo(1,2-d:4,5-d′)bistriazole), containing accessible OMSs within its pore structure, can be transformed through post-synthetic ion exchange into MAF-X27-OH (Co_2_(μ-OH)_2_(bbta)). The resulting framework incorporates both OMSs and hydroxide functionalities while maintaining structural integrity under strongly acidic conditions (0.001 M HCl) and highly alkaline environments (1.0 M KOH) for more than one week. These results further demonstrate the exceptional chemical robustness provided by triazole-based coordination environments [52].

### 4.2. Porosity

Porosity is a critical factor governing CO_2_ adsorption capacity and desorption behavior in triazole-based MOFs. High surface area, large pore volume, and the presence of ultramicroporous channels provide abundant adsorption sites while facilitating efficient molecular transport. In particular, ultramicroporous frameworks are highly advantageous because their confined pore dimensions enable strong overlap between the pore walls and the CO_2_ potential field, resulting in enhanced adsorption enthalpy and improved uptake at low CO_2_ partial pressures [10]. Therefore, precise pore size optimization is essential, as excessively narrow pores can restrict molecular diffusion, whereas oversized pores may weaken host–guest interactions and reduce adsorption selectivity.

Triazole-based MOFs constructed from bis(carboxyphenyl)-1,2,4-triazole ligands coordinated with Cu^2+^, Co^2+^, and Zn^2+^ ions represent an example of how metal selection and ligand geometry collectively regulate framework porosity and CO_2_ adsorption behavior. These materials form isoreticular three-dimensional paddlewheel networks with rtl topology, featuring interconnected microporous channels whose structural characteristics are determined by both linker configuration and metal identity. Depending on the metal center, the resulting frameworks exhibit different degrees of rigidity, with Zn- and Cu-based structures displaying more rigid architectures, whereas Co-based frameworks exhibit greater flexibility. These MOFs possess high intrinsic porosity, with void fractions ranging from approximately 58 to 61%. Their pore dimensions fall within the microporous regime (approximately 540–750 pm), while the pore volumes vary substantially with metal identity and framework flexibility, ranging from ~0.23 to 0.74 cm^3^/g (experimental values).

Among these materials, significant Brunauer–Emmett–Teller (BET) surface areas were obtained mainly for Cu-based frameworks, reaching up to ~1680 m^2^/g, because only these structures provided sufficient accessibility for N_2_ adsorption measurements at 77 K. The resulting porosity strongly influences CO_2_ adsorption behavior, as the pore dimensions are well matched with CO_2_ molecular size, enabling effective diffusion and pore filling. However, CO_2_ uptake is not governed solely by surface area; rather, accessible pore volume and framework flexibility play equally important roles. Flexible frameworks, particularly Co-based MOFs, exhibit stepwise adsorption profiles and gate-opening behavior, whereas more rigid Zn-based frameworks display comparatively limited CO_2_ uptake. Cu-based MOFs achieve a favorable balance between high porosity and stronger host–guest interactions, resulting in enhanced CO_2_ adsorption performance [53].

Furthermore, pore size optimization is essential: pore sizes between 1.0 and 2.0 nm maximize CO_2_ storage capacity by balancing accessible surface area, free volume, and adsorption strength, while too small pores restrict CO_2_ diffusion kinetics and too large pores reduce binding energy and selectivity. The nitrogen-rich triazole ligands further enhance CO_2_ affinity through dipole–quadrupole interactions with the electropositive nitrogen atoms, complementing the porosity effects for superior CO_2_ capture performance.

### 4.3. Functionalization

Functionalization of triazole-based MOFs represents an effective strategy for improving CO_2_ adsorption capacity, selectivity, and performance under practically relevant operating conditions. The incorporation of functional groups, including –NH_2_, –COOH, –OH, alkyl chains, and other polar substituents, into triazole-containing linkers enables precise modulation of the pore environment and strengthens CO_2_ affinity through enhanced electrostatic interactions, dipole–quadrupole interactions, and hydrogen bonding. In these frameworks, the nitrogen atoms of the triazole ring serve as inherent Lewis basic sites that promote CO_2_ adsorption, particularly at low pressures relevant to post-combustion capture from dilute flue gas streams [54,55,56].

A representative example is the pillared metal–organic framework MOF-508, which consists of two-dimensional Zn paddlewheel layers constructed from 1,4-benzenedicarboxylate (bdc) ligands and interconnected by 4,4′-bipyridine (bpy) pillars [57]. In the modified framework MTAF-3, the bpy linker is replaced with a triazole-containing ligand, 4,4′-(2H-1,2,3-triazole-2,4-diyl)dipyridine, while the Zn paddlewheel secondary building units and bdc-based layers remain unchanged. This ligand substitution maintains the original framework topology while introducing electron-rich triazole functionalities into the pore environment. The exposed nitrogen sites associated with the 1,2,3-triazole units provide additional interaction centers for CO_2_ molecules through enhanced quadrupole–dipole and Lewis acid–base interactions. Consequently, MTAF-3 exhibits an approximately threefold enhancement in CO_2_ adsorption capacity compared with MOF-508. This example clearly illustrates that CO_2_ adsorption performance is controlled not only by textural properties such as surface area and pore size, but also by the chemical functionality of the pore surface. In particular, triazole incorporation increases the heat of adsorption (Q_st_) and improves low-pressure CO_2_ affinity without significantly modifying the overall framework topology. Therefore, ligand substitution in pillared MOFs provides an effective approach for optimizing gas adsorption behavior through targeted regulation of pore chemistry rather than complete framework reconstruction [57].

Beyond their influence on porosity, nitrogen-rich channels generated through triazole functionalization further enhance CO_2_ affinity by promoting favorable dipole–quadrupole interactions with electron-rich nitrogen sites. This synergistic contribution of pore architecture and chemical functionality highlights the potential of functionalized triazole-based MOFs for selective CO_2_ capture from challenging gas streams, including flue gas, natural gas, and biogas [56].

However, despite these advantages, functionalization strategies face several important challenges. Precise incorporation of functional groups into triazole-based linkers can be synthetically difficult due to limitations in regioselectivity and site-specific control [58]. In addition, excessive functional group density may lead to pore blocking, reduced accessible surface area, and slower diffusion kinetics, thereby negatively affecting overall CO_2_ uptake.

Although post-synthetic modification (PSM) provides an effective route for introducing additional functionalities into pre-existing frameworks, improper control of the modification process can lead to framework degradation, partial pore collapse, or structural distortion. Such limitations become particularly relevant under realistic flue gas conditions, where the simultaneous presence of CO_2_, H_2_O, SO_x_, and NO_x_ can accelerate structural deterioration and reduce long-term performance [59].

Therefore, an optimal balance between enhanced CO_2_ affinity and preservation of porosity and structural integrity is essential. Rational control over functional group type, density, and distribution within triazole-based frameworks is therefore critical for maximizing adsorption performance under practical conditions.

### 4.4. Incorporation of Active Sites

The introduction of active adsorption sites into triazole-based MOFs represents an effective strategy for improving CO_2_ adsorption capacity, selectivity, and catalytic conversion performance. These active sites may include exposed Lewis acidic metal centers (e.g., Cu^2+^, Zn^2+^, and Ni^2+^), nitrogen-rich triazole functionalities serving as Lewis basic sites, and additional functional groups (such as –NH_2_ and –COOH) that provide enhanced CO_2_ binding through electrostatic interactions and dipole–quadrupole interactions [60]. Gupta and Mandal reported a triazole-functionalized Zn-MOF constructed from a tetracarboxylate ligand and Zn_4_(COO)_6_ secondary building units, resulting in a three-dimensional porous framework with sqc topology and one-dimensional channels. This framework contains two complementary adsorption sites, including exposed Lewis acidic Zn centers and accessible Lewis basic nitrogen atoms from triazole groups. The material exhibits a BET surface area of 314 m^2^/g and a solvent-accessible volume of approximately 18%. The enhanced CO_2_ adsorption performance was primarily attributed to the synergistic contribution of OMSs and triazole functionalities, which promote stronger framework–CO_2_ interactions. In particular, accessible triazole nitrogen sites facilitate favorable quadrupole interactions with CO_2_ molecules, thereby improving adsorption affinity and selectivity [61].

Therefore, CO_2_ adsorption in triazole- based MOFs is governed by a combination of (i) Lewis acidic metal centers that polarize CO_2_, (ii) Lewis basic nitrogen sites that enhance binding, (iii) functional groups that introduce additional polarity, and (iv) pore size optimization that controls diffusion and confinement effects. The synergy between these factors makes triazole-based MOFs highly promising candidates for selective CO_2_ capture and conversion.

## 5. CO_2_ Adsorption Mechanisms in Triazole-Based MOFs

Triazole-based MOFs have emerged as promising porous materials for CO_2_ capture owing to their high thermal and chemical stability, tunable pore environments, and nitrogen-rich coordination characteristics. The incorporation of triazole ligands provides additional adsorption functionalities by promoting CO_2_ interactions through dipole–quadrupole interactions, hydrogen bonding, and coordination with OMSs. One of the fundamental mechanisms governing CO_2_ adsorption in MOFs is the Lewis acid–Lewis base interaction between coordinatively unsaturated metal sites (Lewis acidic centers) and CO_2_ molecules (Lewis bases) [62]. OMSs, such as Ni^2+^ and Zn^2+^, can induce polarization of CO_2_ molecules, thereby strengthening electrostatic interactions and increasing adsorption enthalpy [7,62]. In MOF-74-type frameworks, these exposed metal sites provide a high density of adsorption centers; however, their strong affinity toward water molecules often results in competitive adsorption under humid conditions. In this regard, triazole functionalization can help alleviate moisture-induced performance degradation by enhancing framework stability and introducing additional adsorption pathways that preserve CO_2_ uptake in the presence of water vapor [34].

Beyond metal centers, nitrogen atoms within triazole rings act as intrinsic Lewis basic adsorption sites. These electron-rich nitrogen atoms interact with the electrophilic carbon center of CO_2_ through dipole–quadrupole interactions, which are further supported by hydrogen bonding and electrostatic contributions [55,63]. Computational investigations, including density functional theory (DFT) calculations and Monte Carlo simulations, have revealed that CO_2_ adsorption in triazole-functionalized frameworks involves multiple cooperative noncovalent interactions, including N–H⋯O and C–H⋯O hydrogen bonding as well as electron donor–acceptor (EDA) interactions. The relative strength of these interactions is generally described in the following order: EDA interactions > hydrogen-bond-assisted EDA > C^δ+^⋯N interactions > π–π stacking > conventional hydrogen bonding [64]. The combination of these interactions contributes to the strong initial CO_2_ adsorption observed in various triazole-functionalized MOF systems.

A distinctive characteristic of triazole-based MOFs is their cooperative dual-site adsorption mechanism, which originates from the simultaneous presence of OMSs and triazole nitrogen functionalities. The spatial coexistence of Lewis acidic and Lewis basic adsorption sites enables multipoint interactions with CO_2_ molecules, resulting in stronger binding interactions and improved adsorption capacity compared with single-site adsorption systems. The extent of this synergistic effect depends strongly on framework topology, metal ion identity, and the accessibility of adsorption sites.

Under practical adsorption conditions, moisture represents a critical factor affecting CO_2_ capture performance. Water molecules can competitively coordinate with OMSs or interact with nitrogen-containing functionalities, causing partial blockage of adsorption sites and reducing CO_2_ uptake under humid conditions. However, depending on the adsorption pressure, temperature, and framework hydrophilicity, water may also contribute to alternative adsorption pathways, including the formation of bicarbonate-like species at reactive sites. Therefore, the impact of humidity on CO_2_ adsorption is highly framework-dependent. Strategies such as hydrophobic pore engineering and the development of chemically robust frameworks, including CALF-20, have demonstrated potential for minimizing moisture effects and improving CO_2_ capture performance under humid operating conditions [65,66].

Finally, CO_2_ adsorption selectivity in triazole-based MOFs is primarily determined by the variation in binding energies among different adsorption sites within the framework. Experimental and computational investigations have demonstrated that CO_2_ molecules initially occupy the most energetically favorable adsorption sites, particularly metal-coordinated centers and nitrogen-rich regions. As the pressure increases, additional adsorption sites become progressively accessible, and cooperative adsorbate–adsorbate interactions contribute to further pore filling and increased adsorption capacity. This energy-dependent adsorption behavior provides a general framework for understanding CO_2_ uptake trends across a wide range of triazole-based MOF systems.

## 6. Triazole Based MOFs for CO_2_ Capture

Triazole-based MOFs have attracted considerable attention as promising porous materials for CO_2_ capture owing to their combination of high porosity, remarkable structural stability, and tunable chemical functionality. The integration of triazole ligands into MOF architectures generates nitrogen-rich adsorption environments that promote strong interactions with CO_2_ molecules through dipole–quadrupole interactions, hydrogen bonding, and Lewis acid–base interactions. These synergistic interactions play an important role in improving CO_2_ adsorption capacity and selectivity. In the following sections, the influence of metal centers on CO_2_ adsorption behavior, the role of triazole linker functionalization, and post-synthetic incorporation of triazole functionalities into MOF structures are discussed in detail.

### 6.1. Effect of Metal Centers on CO_2_ Adsorption Performance

Metal centers play a critical role in determining the adsorption environment of triazole-based MOFs, although their effects are closely associated with linker chemistry and pore confinement. In these frameworks, metal nodes contribute not only to the construction of the overall topology and structural stability but also regulate the availability and strength of OMSs, which are key factors governing CO_2_ binding affinity. Based on reported studies, Zn-, Cu-, and Ni-based triazole MOFs exhibit distinct CO_2_ adsorption behaviors, arising from differences in Lewis acidity, coordination flexibility, and interactions with nitrogen-rich triazole ligands.

A representative Zn-based system, ZnATZoxH_2_O, is synthesized hydrothermally from Zn^2+^ ions, 3-amino-1,2,4-triazole (ATZ), and oxalate (C_2_O_4_^2−^) at 453 K over 3 days in water. The structure consists of Zn–triazolate 2D layers pillared by oxalate linkers, forming a rigid 3D ultramicroporous framework with a pore aperture of 3.5 × 2.8 Å^2^ and an effective pore size of ~3.6 Å. Despite the relatively weak Lewis acidity of Zn^2+^, this material delivers CO_2_ uptake of 61.9 cm^3^/g (≈2.76 mmol/g) at 298 K and 1 bar and still 51 cm^3^/g (≈2.27 mmol/g) at 0.1 bar, with a very high Q_st_ of 44.5 kJ/mol. Critically, this performance contradicts the expected metal-centered trend and demonstrates that ultramicropore confinement combined with dense –NH_2_ and oxalate oxygen sites can fully compensate for weaker metal–CO_2_ interactions. The exceptionally high selectivity (CO_2_/N_2_ >10^3^) arises mainly from size exclusion and electrostatic matching rather than Zn^2+^ chemistry, highlighting that the metal centers play a secondary, structural-stabilization role rather than being the dominant adsorption driver [67].

In contrast, Ni-Datz-MOF-74, synthesized solvothermally from Ni^2+^ ions and 3,5-diamino-1,2,4-triazole (Datz), retains the characteristic one-dimensional channel topology of MOF-74, with a BET surface area of 702.55 m^2^/g and a pore volume of 0.27 cm^3^/g. This framework exhibits CO_2_ uptake values of 4.71 mmol/g at 298 K and 0.15 bar and 7.76 mmol/g at 298 K and 1 bar, accompanied by a Q_st_ value of 34.7–49.7 kJ/mol. Ni-Datz-MOF-74 achieves an impressive IAST selectivity of 327 for CO_2_/N_2_ (298 K, 15:85, *v*/*v*). Although Ni^2+^ OMSs contribute significantly to the enhanced adsorption strength, the improved CO_2_ affinity cannot be explained solely by the presence of metal sites. Instead, the superior performance originates from cooperative interactions between Ni-based Lewis acidic OMSs and triazole-derived –NH_2_ functionalities, which promote stronger local polarization of CO_2_ molecules. Notably, partial pore blocking decreases the accessible surface area while simultaneously increasing adsorption energy density, highlighting that chemically accessible adsorption sites can play a more important role than conventional textural parameters alone in determining CO_2_ capture performance [34].

For Cu-based frameworks such as ZSTU-21, synthesized from CuSO_4_·5H_2_O and 3,5-dimethyl-1,2,4-triazole (DMTRZ), Cu(II) centers adopt square-planar and elongated octahedral coordination geometries, resulting in dual OMS environments within a rigid PtS-type three-dimensional framework. Despite possessing a moderate surface area of 677 m^2^/g, ZSTU-21 exhibits a CO_2_ uptake of 62 cm^3^/g (≈2.76 mmol/g) at 298 K and 1 bar, with a Q_st_ value of 28–31 kJ/mol and a CO_2_/N_2_ selectivity of 105. However, the enhanced adsorption performance of this Cu-based framework cannot be attributed solely to the Lewis acidity of Cu centers. Instead, it originates from the combined contribution of heterogeneous adsorption environments arising from dual Cu sites, triazolate nitrogen donors, and confined pore channels. These factors collectively generate multiple weak-to-moderate interaction pathways, allowing Cu-based systems to achieve a balance between adsorption strength and regeneration efficiency [68].

The behavior of Cu/Zn mixed-linker MOFs further highlights the complexity of correlating metal identity with CO_2_ adsorption performance. Although these frameworks contain Cu^2+^ centers with stronger Lewis acidity and Zn^2+^ centers with weaker Lewis acidity, together with 1,3,5-benzenetricarboxylic acid (H_3_BTC) and 3,5-diamino-1,2,4-triazole (DATRZ) linkers, the material exhibits a CO_2_ uptake of 4.06 mmol/g at 298 K and 1 bar, with a Q_st_ value of 15–23 kJ/mol. In this case, hierarchical micro–mesoporosity, characterized by a surface area of 1021 m^2^/g and a pore volume of 0.584 cm^3^/g, plays a dominant role in determining adsorption behavior, whereas metal identity represents a secondary factor affecting diffusion pathways and adsorption site distribution. The relatively low Q_st_ value suggests that, despite the presence of multiple metal centers, limited accessibility and insufficient cooperativity between adsorption sites restrict stronger CO_2_ binding. These findings further demonstrate that metal-centered Lewis acidity alone is not sufficient to ensure superior adsorption performance, and that pore architecture and site accessibility must also be considered [69].

Overall, a general trend in intrinsic CO_2_ affinity can be identified among different metal centers under comparable coordination environments, where Cu(II) typically exhibits stronger Lewis acidity than Ni(II), while Zn(II) generally provides weaker direct interactions with CO_2_ molecules. However, this relationship is significantly influenced by additional structural factors, including ultramicroporous confinement, linker functionality, and cooperative adsorption effects. In many cases, pore confinement and triazole-derived nitrogen sites can compensate for weaker metal–CO_2_ interactions, enabling certain Zn-based frameworks to achieve comparable or even superior CO_2_ adsorption performance relative to Cu-based analogues, particularly under low-pressure conditions. These findings demonstrate that metal identity alone is insufficient for predicting CO_2_ adsorption behavior in triazole-based MOFs. Instead, high-performance adsorbents are achieved through the synergistic integration of metal nodes, triazole functionalities, and carefully engineered pore environments, which collectively generate multi-site adsorption landscapes that favor both high capacity and enhanced selectivity.

### 6.2. Influence of Triazole Linker Functionalization on CO_2_ Adsorption Performance

#### 6.2.1. Pure Metal-Triazolate Frameworks

Pure metal–triazolate frameworks provide an excellent platform for understanding the intrinsic role of ligand chemistry in CO_2_ adsorption, as triazolate ligands serve as the only structural components without the contribution of auxiliary linkers. This structural simplicity enables direct investigation of how subtle electronic and steric modifications of the triazole ring influence CO_2_ adsorption energetics, selectivity, and capacity. Nevertheless, the absence of additional functional components also reveals an important limitation: adsorption behavior in these systems can be highly sensitive to minor chemical modifications, indicating that pore architecture alone cannot adequately predict CO_2_ affinity.

**Figure 3:**
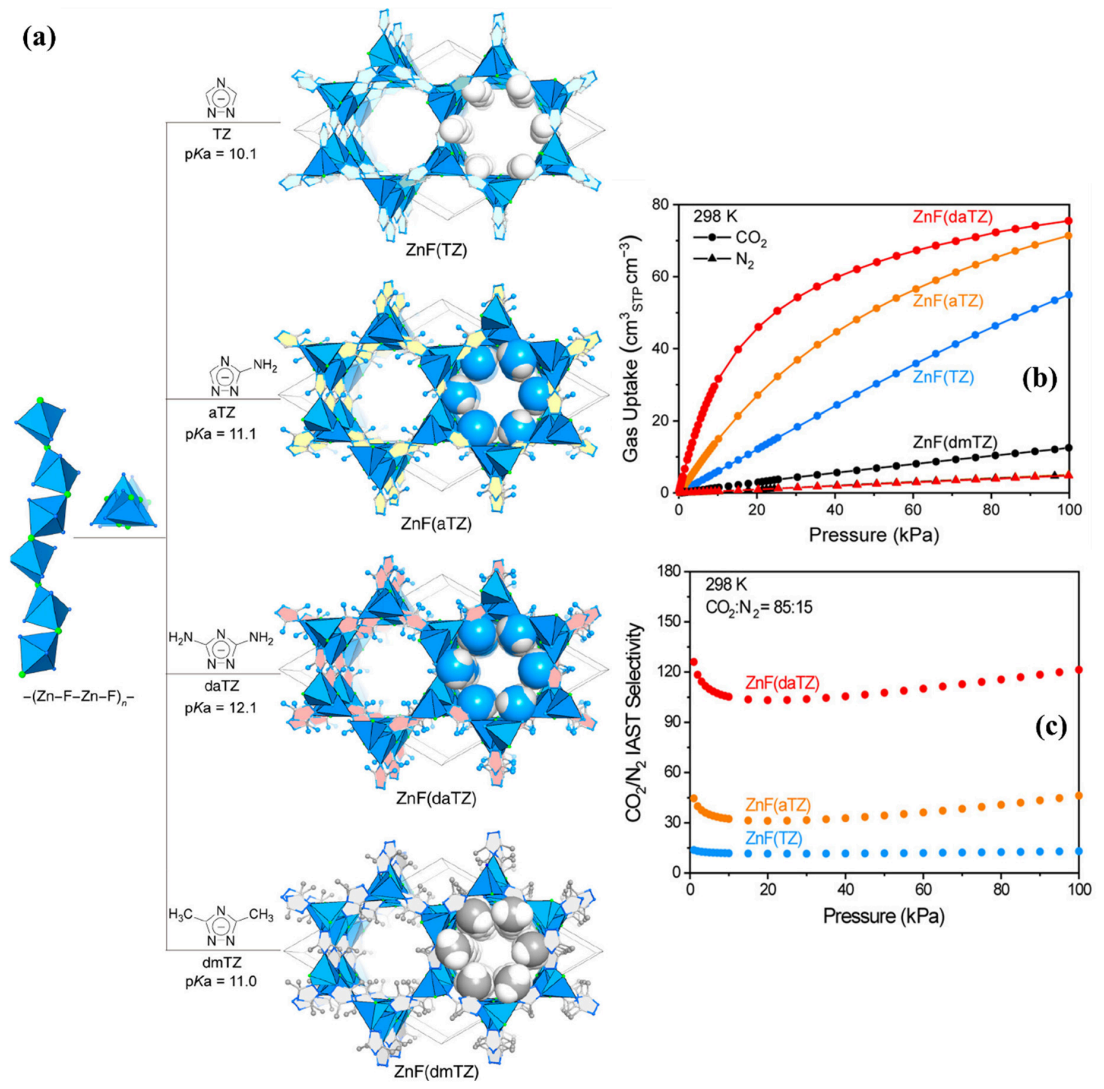
(**a**) Crystal structures of ZnF(TZ), ZnF(aTZ), ZnF(daTZ), and ZnF(dmTZ); (**b**) CO_2_ and N_2_ adsorption isotherms at 298 K over the pressure range of 0–1 bar; (**c**) IAST selectivity of CO_2_/N_2_ for a mixed gas composition of CO_2_/N_2_ (*v*/*v* = 15:85) at 298 K. Reproduced from Ref. [70] with permission from ACS, 2020.

The ZnF(TZ), ZnF(aTZ), ZnF(daTZ), and ZnF(dmTZ) series (where aTZ = 3-amino-1,2,4-triazolate, daTZ = 3,5-diamino-1,2,4-triazolate, and dmTZ = 3,5-dimethyl-1,2,4-triazolate) represents a valuable example for establishing direct structure–property relationships. These frameworks are constructed from rod-like (Zn–F–Zn–F)_n_ secondary building units connected by three-connected triazolate ligands, generating honeycomb-type one-dimensional channels. Because all members retain the same overall topology, the influence of functional group modification on CO_2_ adsorption can be directly evaluated (Figure 3a) [70]. The parent ZnF(TZ) framework exhibits a surface area of 613 m^2^/g and a pore volume of 0.213 cm^3^/g, but shows the lowest CO_2_ uptake within this series (0.27 mmol/g at 1.5 mbar), with a CO_2_/N_2_ selectivity of 13 and a Q_st_ value of 23.6–24 kJ/mol. Importantly, this relatively weak adsorption performance despite comparable ultramicroporosity demonstrates that confined pore dimensions alone are insufficient to achieve strong CO_2_ affinity without appropriate chemical interactions (Figure 3b,c). Introducing amino functionality significantly alters the adsorption behavior. ZnF(aTZ), despite exhibiting reduced surface area (573 m^2^/g) and pore volume (0.168 cm^3^/g), achieves approximately twice the CO_2_ uptake (0.56 mmol/g at 1.5 mbar) and substantially higher selectivity (~46), accompanied by an increased Q_st_ value of 32.3 kJ/mol. This improvement highlights that –NH_2_ incorporation can compensate for reduced textural properties by providing additional electrostatic interactions and hydrogen-bonding sites for CO_2_ adsorption (Figure 3b,c). The effect becomes more pronounced in ZnF(daTZ), where the introduction of two amino groups results in the highest CO_2_ uptake (0.96 mmol/g at 1.5 mbar) and selectivity (120), despite further reductions in surface area (479 m^2^/g) and pore volume (0.119 cm^3^/g). The increase in Q_st_ to approximately 33 kJ/mol indicates stronger CO_2_-framework interactions and enhanced adsorption energetics. However, the limited additional improvement at higher functional group density suggests that excessive functionalization within ultramicropores may introduce steric constraints and reduce the benefit of further chemical modification (Figure 3b,c). In contrast, ZnF(dmTZ), containing hydrophobic methyl substituents, exhibits lower CO_2_ uptake and selectivity compared with amino-functionalized analogues. Although this framework maintains comparable textural characteristics, the –CH_3_ groups do not provide favorable CO_2_ binding interactions and may reduce framework polarity while partially restricting access to adsorption sites. Consequently, weaker CO_2_ affinity is observed. Overall, the adsorption trend follows the order ZnF(daTZ) > ZnF(aTZ) > ZnF(TZ), demonstrating that CO_2_ capture performance in triazolate-based MOFs is primarily governed by chemical functionality rather than surface area or pore volume alone. While textural parameters determine the available adsorption space, the nature, density, and distribution of functional groups ultimately control adsorption strength, selectivity, and thermodynamic behavior. Amino-functionalization enhances CO_2_ capture through cooperative Lewis acid–base interactions and hydrogen bonding, whereas hydrophobic substitution reduces adsorption efficiency [70].

A contrasting example is MAF-66, a 3D diamondoid (dia) triazolate framework constructed from Zn^2+^ ions coordinated with deprotonated 3-amino-1,2,4-triazolate ligands. This framework forms a non-interpenetrated network containing interconnected channels with dimensions of 3.2 × 6.8 Å^2^. Unlike the ZnF-series frameworks, where adsorption behavior is primarily controlled by localized functional group effects, MAF-66 exhibits a distinct adsorption mechanism despite its high porosity, with a surface area of 1014 m^2^/g and a pore volume of 0.43 cm^3^/g. MAF-66 achieves CO_2_ uptake values of 6.26 mmol/g (140 cm^3^(STP)/g) at 273 K and 1 atm and 4.41 mmol/g (99 cm^3^(STP)/g) at 298 K and 1 atm. The corresponding Q_st_ increases from 26.0 kJ/mol at zero coverage to 30.2 kJ/mol at higher loading, indicating progressively strengthened CO_2_–framework interactions during adsorption. Notably, the exceptionally high CO_2_/N_2_ selectivity values of 403 at 273 K and 225 at 298 K cannot be attributed solely to surface area or the presence of isolated functional groups. Instead, the outstanding separation performance originates from the combination of a high density of uncoordinated nitrogen sites (17.3 mmol/g), strong pore confinement effects, and partial framework flexibility, which together promote cooperative adsorption behavior rather than independent site-specific interactions. This behavior differentiates MAF-66 from ZnF-based systems, where adsorption is mainly governed by the chemical contribution of individual functional groups. In contrast, MAF-66 demonstrates that collective adsorption effects arising from high site density, confined pore environments, and framework topology can play a dominant role in achieving exceptional CO_2_ selectivity and uptake performance [71].

A critical comparison of these two framework families reveals that CO_2_ capture enhancement in pure triazolate MOFs can proceed through two fundamentally different mechanisms. In low-dimensional ZnF-type frameworks, adsorption behavior is primarily dictated by local electronic modulation of the triazolate ligand. In these systems, amino functionalization enhances CO_2_ binding through additional Lewis acid–base interactions and hydrogen bonding, whereas methyl substitution decreases adsorption affinity by lowering framework polarity and limiting access to adsorption sites. In contrast, 3D dia frameworks such as MAF-66 exhibit a different adsorption mechanism, where performance is not primarily determined by individual functional group modifications but rather by the overall spatial arrangement of nitrogen-rich adsorption sites, ultramicroporous confinement, and cooperative host–guest interactions. These differences highlight an important consideration in structure–property analysis of triazolate-based MOFs: direct comparison of CO_2_ uptake or selectivity values across different frameworks can be misleading if factors such as topology, pore dimensionality, and adsorption site density are not simultaneously considered. The same functional group can therefore produce substantially different adsorption behavior depending on the surrounding framework architecture and local adsorption environment.

#### 6.2.2. Mixed Linker Triazolate MOFs

Mixed-linker triazolate-based MOFs represent a highly versatile family of CO_2_ adsorbents, where triazolate ligands are combined with secondary inorganic (carbonate and oxalate) or organic (dicarboxylate, squarate, and azobenzene carboxylate) linkers to create chemically diverse and structurally confined adsorption environments. In these systems, CO_2_ capture performance cannot be adequately described by conventional textural parameters such as surface area or pore volume alone. Instead, adsorption behavior results from the combined effects of ultramicroporous confinement, spatial distribution of adsorption sites, and linker-induced modulation of framework polarity and electrostatic interactions. Therefore, the most effective mixed-linker frameworks are generally those that achieve an appropriate balance between confinement-enhanced physisorption and chemically assisted CO_2_ binding, rather than relying exclusively on either mechanism.

**Figure 4:**
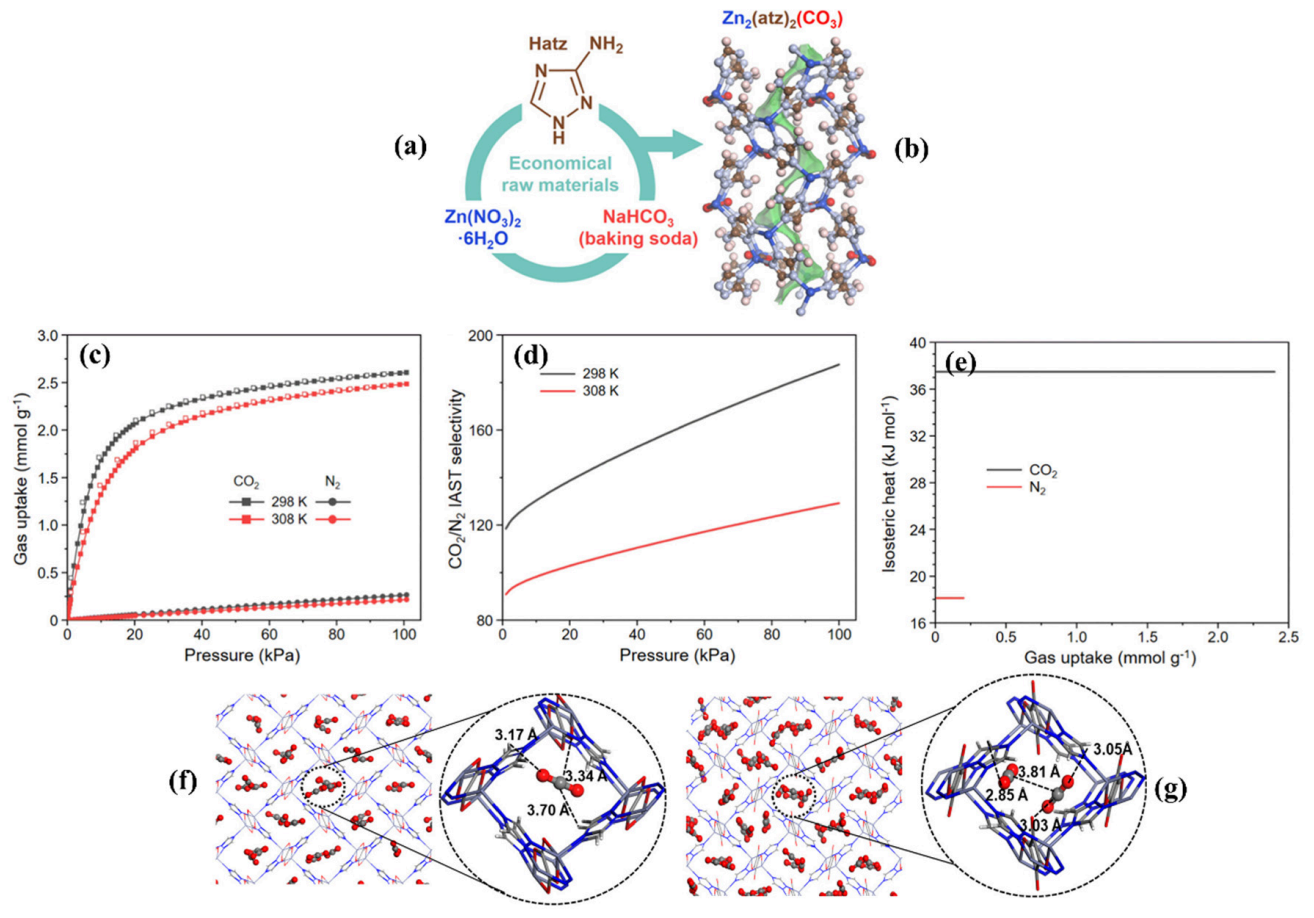
(**a**) Formation reaction involving Zn^2+^ ions, sodium bicarbonate, and 3-amino-1,2,4-triazole (Hatz) ligands. (**b**) The resulting 3D framework, where Zn–atz layers are pillared by carbonate (CO_3_^2−^) ligands. These Zn–atz layers are arranged in an AAmAAm stacking sequence, in which the subscript *m* denotes a mirror symmetry along the *b*-axis. (**c**) CO_2_ and N_2_ adsorption isotherms measured at 298 and 308 K, with CO_2_ desorption branches shown using open symbols. (**d**) IAST-calculated CO_2_/N_2_ selectivity for a mixed gas composition (CO_2_/N_2_ = 15/85, *v*/*v*) at 298 and 308 K. (**e**) Q_st_ values for CO_2_ and N_2_ derived via analytical differentiation. Reproduced from Ref. [72] with permission from ACS, 2024. (**f**,**g**) Snapshots from single-component Monte Carlo simulations at 0.15 bar showing preferred CO_2_ adsorption sites and characteristic interaction distances in CALF-20 and SquCALF-20, respectively. For clarity, one unit cell of each framework is shown in the inset, where CALF-20 accommodates one CO_2_ molecule per unit cell, while SquCALF-20 accommodates two CO_2_ molecules per unit cell under the same conditions. Reproduced from Ref. [77] with permission from ACS, 2024.

Zn_2_(atz)_2_(CO_3_) (atz = 3-amino-1,2,4-triazolate) provides an illustrative example of this design strategy. This framework is synthesized through a green aqueous route using Zn^2+^ precursors and sodium bicarbonate, where in situ generated carbonate ions act as rigid inorganic pillars connecting Zn–triazolate layers and producing a robust three-dimensional pillared architecture (Figure 4a,b) [72]. Despite its moderate BET surface area of 545 m^2^/g, the framework contains highly confined ultramicroporous channels with a pore-limiting diameter of ~3.52 Å and cavity dimensions of ~4.69 Å. These restricted pores enable a CO_2_ uptake of 1.9 mmol/g at 298 K and 15 kPa, together with a high CO_2_/N_2_ IAST selectivity of ~90–190 (≈188 at 298 K, 100 kPa), demonstrating that effective molecular discrimination can be achieved without exceptionally high surface area. The relatively high isosteric heat of adsorption (Q_st_ ≈ 37.5 kJ/mol), compared with that of N_2_ (18.1 kJ/mol), reflects preferential stabilization of CO_2_ molecules within the confined adsorption environment. This behavior originates from the combined contributions of Lewis acid–base interactions involving triazolate –NH_2_ groups, hydrogen bonding with framework N–H/C–H sites, and electrostatic effects generated by carbonate pillars, which together create a polar environment favorable for CO_2_ adsorption [72].

Another representative mixed-linker system is NICS-24, synthesized from Zn(NO_3_)_2_·3H_2_O and 3,5-diamino-1,2,4-triazolate under hydrothermal or scalable reflux conditions. This framework adopts a three-dimensional sqc topology consisting of oxalate- and triazolate-bridged Zn chains, generating dual ultramicroporous channels with dimensions of approximately 3.5 and 5.0 Å [73]. Although NICS-24 possesses a relatively low BET surface area (274 m^2^/g), it achieves a CO_2_ uptake of ~3.0 mmol/g at 298 K and 1 bar and maintains an uptake of ~0.7 mmol/g at 2 mbar, exceeding the performance of CALF-20 and CALF-15 under dilute CO_2_ conditions. However, this enhanced low-pressure capture capability is associated with a very high Q_st_ value (~68 kJ/mol), indicating a stronger contribution from amine–CO_2_ interactions approaching chemisorption-like behavior. Consequently, NICS-24 represents an extreme case of the adsorption–regeneration trade-off, where strong binding interactions improve trace CO_2_ capture but may increase the energy requirement for regeneration. The enhanced selectivity (~8-fold for CO_2_/N_2_ and ~30-fold for CO_2_/O_2_) is attributed to intensified hydrogen-bonding interactions within the ultraconfined channels, highlighting that low-concentration CO_2_ capture is primarily controlled by adsorption-site chemistry and interaction strength rather than porosity parameters alone [73].

ZnDatzBdc is a flexible Zn(II)-based pillared-layer MOF constructed from 3,5-diamino-1,2,4-triazolate (Datz) and 1,4-benzenedicarboxylate (Bdc), exhibiting reversible closed-to-open pore transformation associated with hydrogen-bond rearrangement and phenyl linker rotation [74]. The activated framework possesses a BET surface area of 303 m^2^/g and a pore volume of 0.11 cm^3^/g; however, it demonstrates notable CO_2_ adsorption performance, with uptake values of 2.89 mmol/g at 273 K and 2.05 mmol/g at 298 K, accompanied by CO_2_/N_2_ selectivity values of 107 (273 K) and 129 (298 K). Unlike rigid porous frameworks, the adsorption behavior of ZnDatzBdc is strongly coupled with structural dynamics. The adsorption energetics change during the framework transition, with Q_st_ increasing from ~26–29 kJ/mol in the closed state to ~32.5–34.5 kJ/mol in the open state, indicating that CO_2_ adsorption can induce structural activation and create more favorable binding environments. This dynamic pore-opening behavior demonstrates the potential of flexible MOFs to enhance working capacity by adapting their pore structure during adsorption. Nevertheless, the presence of framework flexibility also requires careful evaluation, particularly regarding long-term structural stability and performance retention during repeated cycling under realistic conditions, including humid environments [74].

Zn_2_(C_2_O_4_)(C_2_N_4_H_3_)_2_·(H_2_O)_0_._5_ represents another example of a mixed-linker triazolate framework, consisting of Zn^2+^ nodes, oxalate linkers, and 3-amino-1,2,4-triazolate ligands within a three-dimensional α-Po topology. The framework contains ultramicroporous channels of approximately 3.5 × 4.0 Å and exhibits a relatively high surface area of 782 m^2^/g [75]. It achieves CO_2_ uptakes of 4.35 mmol/g at 273 K and 1.2 bar and 3.78 mmol/g at 293 K, with Q_st_ values decreasing from 40.8 kJ/mol at zero coverage to 38.6 kJ/mol at higher loading. Although this material provides a high CO_2_ uptake compared with several related frameworks, its CO_2_-selective adsorption behavior highlights that adsorption capacity alone does not necessarily determine separation efficiency. Instead, its adsorption behavior arises from a combination of ultramicropore confinement, amine–CO_2_ quadrupolar interactions, and cooperative adsorbate–adsorbate interactions at higher loading levels, emphasizing the importance of collective adsorption phenomena in densely confined pore systems [75].

CALF-20 ([Zn_2_(triazolate)_2_(oxalate)]) is considered a benchmark triazolate–oxalate framework because of its combination of ultramicroporosity, adsorption selectivity, and environmental stability. This material possesses narrow channels (2.73–3.11 Å) with a BET surface area of 528 m^2^/g and exhibits CO_2_ uptake of 4.07 mmol/g at 293 K and 1.2 bar, together with an exceptionally high CO_2_/N_2_ IAST selectivity of ~230 and Q_st_ values of approximately 39 kJ/mol [76]. A particularly important feature of CALF-20 is its excellent tolerance toward humidity, maintaining adsorption performance under relative humidity levels up to ~40%. This behavior originates from the limited interaction between water molecules and the framework, resulting from the absence of highly hydrophilic coordination sites. Mechanistic studies indicate that CO_2_ adsorption is dominated by dispersion interactions (~85%), with CO_2_ molecules preferentially stabilized near pore centers (~3.03 Å), whereas water molecules form non-competitive hydrogen-bonded clusters. This separation between CO_2_ adsorption and water affinity represents an unusual design advantage, where moderate interactions combined with optimized spatial confinement enable practical gas separation performance [76].

SquCALF-20, an isoreticular derivative of CALF-20, further demonstrates the impact of subtle linker modification on adsorption behavior. In this framework, oxalate linkers are replaced by squarate groups while preserving the overall topology, resulting in enhanced pore polarity and improved electronic complementarity [77]. Although the porosity changes only slightly (pore volume ~0.35 vs ~0.32 cm^3^/g), CO_2_ uptake increases to ~3.6 mmol/g at 0.15 bar and ~4.9 mmol/g at 1 bar, while CO_2_/N_2_ selectivity rises significantly to ~500 compared with ~180 for CALF-20. The Q_st_ value remains within an optimal physisorption range (~38 kJ/mol), suggesting that the improved performance does not originate from excessively strong binding but rather from optimized electrostatic potential distribution and more efficient CO_2_ packing within ultramicropores (~2.9 Å limiting aperture). This example highlights that targeted linker modification can be more effective than increasing pore volume when the objective is to improve selective CO_2_ capture [77].

A different design strategy was reported by Zou et al., who developed a mixed-ligand Zn(II) anionic MOF incorporating azobenzene tetracarboxylate (ABTC^4−^) and 1,2,4-triazolate ligands. The framework contains two distinct secondary building units, including Zn-paddlewheel [Zn_2_(COO)_4_] clusters and Zn–triazolate [Zn_2_(Tz)_2_(COO)_4_] units [78]. This three-dimensional structure exhibits a BET surface area of 976 m^2^/g. The CO_2_ adsorption amounts were 92.1 cm^3^ (STP)/g (≈4.11 mmol/g) at 273 K and 46.4 cm^3^ (STP)/g (≈2.07 mmol/g) at 298 K. However, despite its high porosity and structural complexity, the CO_2_/N_2_ selectivity remains relatively low (~5.1–5.5), accompanied by a Q_st_ value of approximately 25 kJ/mol. These results demonstrate that increased surface area and structural diversity alone are insufficient to achieve effective gas separation. In this case, adsorption is mainly influenced by electrostatic interactions associated with the anionic framework and pore confinement, while the lack of highly specific CO_2_-binding sites limits overall selectivity [78].

Collectively, mixed-linker triazolate MOFs demonstrate that CO_2_ adsorption performance is controlled by the combined effects of pore confinement, chemical functionality, and framework polarity rather than by any single structural parameter. Within these materials, ultramicroporous environments provide the fundamental basis for molecular discrimination, whereas linker-derived functional groups regulate adsorption energetics and selectivity through localized electrostatic interactions and hydrogen bonding. An important design consideration is the balance between adsorption strength and regeneration efficiency. In many mixed-linker triazolate frameworks, optimal performance is achieved when Q_st_ values remain within a moderate range (~30–40 kJ/mol), providing sufficient CO_2_ affinity while avoiding excessive energy requirements during regeneration. Frameworks with weaker interactions generally exhibit limited selectivity, whereas excessively strong binding can compromise practical applicability by increasing regeneration penalties. Therefore, future development of mixed-linker triazolate MOFs should focus on the cooperative optimization of pore confinement and chemical heterogeneity rather than pursuing maximum porosity or binding strength alone.

#### 6.2.3. Post-Synthetic Triazole Incorporation

Post-synthetic triazole incorporation represents a highly effective strategy for enhancing CO_2_ adsorption in pre-formed MOFs without compromising their inherent crystallinity, topology, or thermal stability. In this approach, nitrogen-rich triazole functionalities are introduced into existing porous hosts via covalent grafting, solvent-assisted linker exchange, or partial linker functionalization. This modification increases framework polarity and introduces additional Lewis basic adsorption sites, thereby strengthening interactions with quadrupolar CO_2_ molecules while preserving the overall structural integrity of the parent framework. From a critical perspective, the performance enhancement is primarily governed by the balance between increased adsorption site density and the inevitable reduction in accessible porosity caused by partial pore occupation.

**Figure 5:**
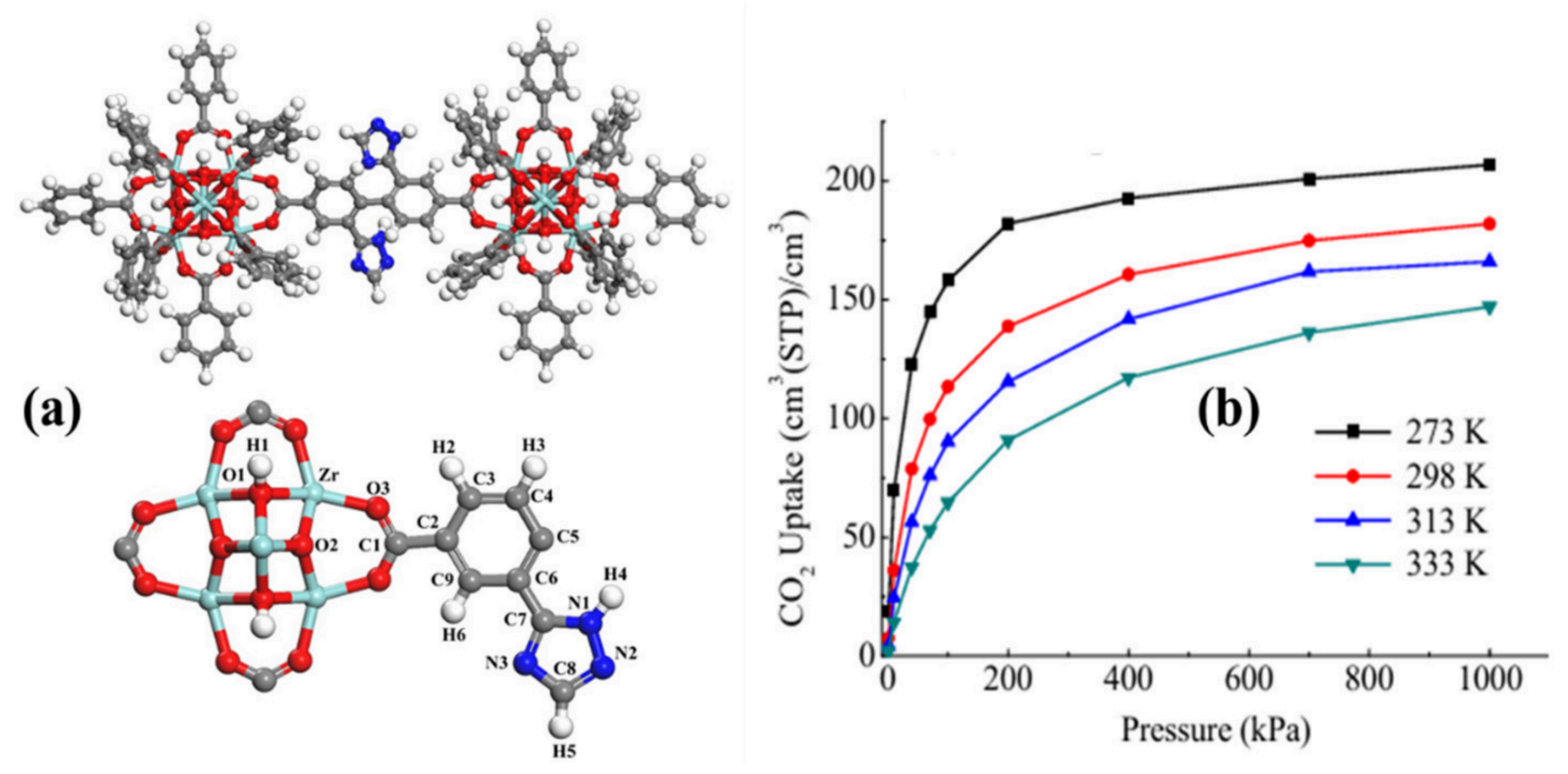
(**a**) Cluster model employed to compute the atomic charges of Triazole@UiO-67, with hydrogen atoms added at the boundaries to reduce edge effects. (**b**) CO_2_ adsorption isotherms of Triazole@UiO-67 measured up to 1000 kPa at 273 K, 298 K, 313 K, and 333 K. Reproduced from Ref. [79] with permission from ACS, 2019.

Triazole-functionalized UiO-67 (Triazole@UiO-67) is obtained through post-synthetic incorporation of triazole heterocycles into the Zr-based UiO-67 framework while fully retaining its fcu topology (Figure 5a). The material exhibits a high BET surface area of 1039 m^2^/g and a pore volume of 0.475 cm^3^/g, indicating that functionalization does not significantly compromise the intrinsic porosity of the parent structure. CO_2_ uptake reaches 113.4 cm^3^(STP)/cm^3^ (≈4.2 mmol/g, calculated using a density of 1.2 g/cm^3^) at 298 K and 100 kPa and increases to 182 cm^3^(STP)/cm^3^ at 1000 kPa, demonstrating strong pressure-dependent adsorption behavior (Figure 5b). The isosteric heat of adsorption (Q_st_ = 37.34 kJ/mol) lies within an optimal range for reversible physisorption, ensuring a favorable balance between uptake capacity and regeneration energy. Molecular simulations further reveal CO_2_–triazole interaction energies of approximately −15.22 kJ/mol, while SAPT decomposition indicates that electrostatic interactions account for nearly 70% of the total adsorption energy. Mechanistically, the improved performance originates from the introduction of triazole nitrogen sites, which enhance framework polarity and induce mild pore constriction, thereby reinforcing confinement-driven adsorption enhancement [79].

Post-synthetic linker modification has also been explored in benzotriazole-functionalized ZIF-8 through solvent-assisted linker exchange, where partial replacement of imidazolate linkers introduces additional nitrogen-rich adsorption sites into the pore environment. Although this modification decreases the BET surface area from 1744 m^2^/g to 1210 m^2^/g and reduces the pore volume from 0.60 cm^3^/g to 0.48 cm^3^/g, the CO_2_ uptake increases from 37.02 cm^3^/g (≈1.65 mmol/g) to 39.88 cm^3^/g (≈1.77 mmol/g) at 273 K and 1.2 bar. The enhanced adsorption is accompanied by an increase in Q_st_, indicating stronger framework–CO_2_ interactions. Rather than originating from increased porosity, this improvement is attributed to the combined effects of benzotriazole-derived polar nitrogen sites, partial pore narrowing, and the generation of heterogeneous high-energy adsorption regions that preferentially stabilize CO_2_ molecules. Furthermore, the modified ZIF-8 exhibited enhanced CO_2_/N_2_ selectivity, with values of 10.47 and 13.70 at 273 and 298 K, respectively, compared with 7.11 and 8.80 for pristine ZIF-8 under identical conditions. These results demonstrate that nitrogen-rich post-synthetic functionalization can simultaneously improve CO_2_ affinity and gas separation performance despite partial reductions in accessible porosity [80].

A similar strategy has been applied to MIL-68(In)-NHTr, derived from MIL-68(In)-NH_2_ through incorporation of triazole functionalities into the organic linker backbone while maintaining the characteristic one-dimensional channel structure of the parent framework. The introduction of triazole groups decreases the BET surface area from 750 m^2^/g to 405 m^2^/g due to partial pore occupation; however, permanent microporosity and thermal stability up to 390 °C are retained. Interestingly, despite the reduction in textural properties, CO_2_ uptake increases significantly to 83.5 cm^3^/g (≈3.7 mmol/g) at 273 K compared with 52.6 cm^3^/g for the non-triazolated analogue. The Q_st_ value of 32.5 kJ/mol indicates moderately strong yet reversible physisorption, while temperature-programmed desorption confirms the presence of medium-strength Lewis basic sites (~554 K) associated with triazole nitrogen atoms. These sites enhance CO_2_ affinity through electrostatic interactions and quadrupole-based interactions [81].

Overall, post-synthetic triazole incorporation improves CO_2_ capture performance by increasing framework polarity, introducing additional Lewis basic adsorption sites, and modifying local confinement effects while preserving the fundamental network topology. Nevertheless, these approaches involve an inherent structure–property trade-off, where enhanced adsorption affinity is often accompanied by reduced accessible pore volume. Therefore, the effectiveness of post-synthetic modification depends on achieving an appropriate balance between functional site density and molecular accessibility. The most successful examples are those that introduce energetic heterogeneity and additional CO_2_ binding sites without significantly restricting diffusion pathways or compromising pore accessibility.

A comprehensive comparison of representative triazole-based MOFs and benchmark CO_2_ adsorbents is provided in Table 2, highlighting key performance parameters including CO_2_ uptake, CO_2_/N_2_ selectivity, Q_st_, and dominant adsorption mechanisms across different structural families. The comparison demonstrates that triazole-based MOFs constitute a chemically diverse and structurally tunable class of adsorbents that can achieve a balanced combination of moderate-to-high CO_2_ uptake, enhanced low-pressure selectivity, and favorable adsorption energetics. Importantly, their performance cannot be predicted solely from surface area or pore volume; instead, it arises from the combined contributions of nitrogen-rich triazole functionalities, ultramicroporous confinement, and, in selected cases, open metal sites or mixed-linker architectures. Compared with conventional MOF families, triazole-based frameworks occupy an intermediate but highly competitive position between strongly interacting OMS-dominated materials, such as MOF-74-type systems, and highly stable but less chemically interactive porous frameworks such as ZIFs. This balance provides opportunities for achieving improved selectivity while maintaining acceptable regeneration requirements.

From a broader design perspective, the performance trends summarized in Table 1 indicate that CO_2_ adsorption in triazole-based MOFs is controlled by topology-dependent mechanisms. In low-dimensional or rod-SBU triazolate frameworks, adsorption enhancement is mainly achieved through local chemical modification, particularly amino functionalization, which strengthens electrostatic interactions and hydrogen bonding with CO_2_. In contrast, three-dimensional and mixed-linker frameworks rely more strongly on cooperative effects involving multiple adsorption sites, including dual secondary building units, charged framework environments, and hierarchical pore structures. Across these systems, ultramicroporous confinement consistently emerges as a critical factor that enhances adsorption efficiency, even in frameworks with relatively modest surface areas. However, the balance between adsorption strength and regeneration energy remains a central challenge, particularly for highly amine-functionalized or strongly confined systems, where excessive binding interactions may limit long-term practical application under cyclic capture conditions.

## 7. Comparative Performance of Selected MOF Families for CO_2_ Capture

Compared with conventional MOF families, triazole-based MOFs occupy a distinct and increasingly competitive position in CO_2_ capture due to their ability to simultaneously integrate optimized pore architectures with chemically active adsorption sites. In contrast to surface-area-driven frameworks such as MOF-5-type materials, where high gravimetric uptake is often associated with adsorption at relatively high pressures and limited low-pressure affinity, triazole-based frameworks provide nitrogen-rich coordination environments combined with ultramicroporous confinement, enabling stronger and more selective CO_2_ interactions under near-ambient conditions. MOF-74-type materials represent another important class, where high CO_2_ uptake originates primarily from the presence of exposed OMSs. Although these sites provide strong CO_2_ binding and high adsorption enthalpies, their strong affinity toward polar molecules, particularly water, can result in competitive adsorption under humid conditions and increase the energy demand required for regeneration. ZIFs, despite their excellent thermal and chemical stability, generally contain less polar pore environments, which can limit intrinsic CO_2_ affinity unless additional functionalization strategies are applied. Similarly, UiO-type frameworks are recognized for their exceptional hydrothermal and chemical robustness; however, their relatively weaker host–guest interactions often lead to moderate CO_2_ selectivity compared with more chemically functionalized porous systems.

Triazole-based MOFs combine several desirable characteristics, including abundant Lewis basic nitrogen sites, tunable ultramicroporous environments, and, in selected cases, cooperative adsorption effects arising from mixed-linker strategies or inorganic pillar incorporation. Consequently, many triazole-containing frameworks exhibit enhanced low-pressure CO_2_ adsorption, high CO_2_/N_2_ selectivity, and intermediate adsorption enthalpies that provide a favorable balance between capture efficiency and regeneration requirements. Overall, their performance is not determined by surface area alone but results from the synergistic contribution of pore confinement, nitrogen-rich chemical environments, and heterogeneous adsorption sites.

Table 3 summarizes representative MOF families for CO_2_ capture, highlighting key performance descriptors, dominant adsorption mechanisms, and inherent structural trade-offs under near-ambient conditions.

From a broader perspective, the comparative analysis indicates that achieving optimal performance across all CO_2_ capture parameters remains challenging for any single MOF family. In practice, adsorption capacity, selectivity, adsorption strength, regeneration energy, and stability under operating conditions are closely interconnected, and improvements in one parameter often involve compromises in others. Triazole-based MOFs represent a balanced class within this performance landscape, where moderate adsorption enthalpies are combined with enhanced low-pressure CO_2_ affinity and improved selectivity through nitrogen-rich coordination environments and ultramicroporous confinement.

Compared with OMS-dominated materials such as MOF-74, triazole-based frameworks generally provide weaker but more reversible CO_2_ interactions, which can reduce regeneration requirements and improve tolerance toward humid gas streams. At the same time, their chemically active nitrogen sites and confined pore environments often enable higher low-pressure CO_2_ selectivity than less polar frameworks, including many ZIF- and UiO-type materials. Similarly, compared with highly porous but relatively weakly interacting MIL-type MOFs, triazole-based systems achieve stronger thermodynamic discrimination toward CO_2_ despite, in some cases, possessing lower overall surface areas.

Therefore, the major advantage of triazole-based MOFs does not originate from maximizing surface area or pore volume alone. Rather, their practical potential arises from the rational integration of framework robustness, accessible adsorption sites, and appropriately balanced adsorption energetics. This combination provides a favorable platform for achieving efficient and reversible CO_2_ capture under realistic cyclic operating conditions.

## 8. Effect of Structural Characteristics on CO_2_ Capture in Triazole-Based MOFs

The CO_2_ adsorption performance of triazole-based MOFs is determined by the combined influence of textural characteristics, framework chemistry, and the nature and accessibility of adsorption sites. Although high surface area and pore volume provide the physical space required for gas accommodation, they do not solely dictate adsorption efficiency. Parameters such as ultramicroporous confinement, nitrogen-rich triazole functionalities, coordinatively unsaturated metal sites, and functional groups within the pore environment play a more direct role in controlling CO_2_ affinity, selectivity, and low-pressure adsorption behavior. Therefore, the most effective triazole-based MOFs are not necessarily those with the largest pore volume or surface area, but rather those in which accessible porosity is carefully balanced with favorable host–guest interactions.

At low CO_2_ partial pressures, adsorption is primarily controlled by high-affinity sites, including Lewis basic nitrogen atoms and exposed metal centers, which promote strong dipole–quadrupole and electrostatic interactions with CO_2_ molecules. As the pressure increases and the high-energy sites become progressively occupied, additional contributions from pore filling and adsorbate–adsorbate interactions become increasingly important. Temperature also strongly influences adsorption behavior; elevated temperatures generally decrease CO_2_ uptake, while higher Q_st_ values indicate stronger interactions and enhanced selectivity but may also increase the energy required for regeneration. Consequently, practical triazole-based MOFs require an appropriate balance between adsorption strength, porosity, and regenerability rather than maximizing any single parameter.

Mechanistically, CO_2_ adsorption within triazole-based frameworks involves multiple cooperative interactions, including Lewis acid–base interactions, dipole–quadrupole forces, hydrogen bonding, van der Waals interactions, and pore confinement effects. The electron-rich nitrogen atoms of triazole ligands provide intrinsic Lewis basic sites, whereas coordinatively unsaturated metal centers contribute complementary Lewis acidic interactions. The coexistence of these adsorption motifs generates chemically heterogeneous environments containing sites with different binding energies. Computational and experimental studies suggest that CO_2_ molecules initially occupy the strongest adsorption sites at low coverage, followed by progressive filling of less favorable sites as loading increases. This hierarchical adsorption behavior emphasizes that the performance of triazole-based MOFs is primarily governed by the spatial distribution, accessibility, and strength of adsorption sites rather than surface area alone.

## 9. Challenges and Future Perspectives

Despite substantial progress in the development of triazole-based MOFs for CO_2_ capture, several challenges still limit their practical and large-scale implementation. One of the main issues is the lack of well-defined and generalizable structure–property relationships. Although individual descriptors such as pore size, surface area, metal centers, and triazole functionalization have each been associated with CO_2_ adsorption performance, their combined and sometimes competing effects remain insufficiently understood. This makes the rational design of materials with predictable adsorption behavior particularly challenging. Integrating experimental studies with computational modeling and data-driven approaches could provide valuable insights to address this limitation.

Another major challenge is balancing CO_2_ adsorption strength with regenerability. Many high-performing triazole-based MOFs exhibit relatively high Q_st_ values due to strong interactions between CO_2_ molecules and nitrogen-rich triazole sites or open metal centers. While these interactions enhance uptake capacity and selectivity at low pressures, they can also increase the energy required for regeneration. Therefore, the development of materials with moderate binding energies that preserve high performance while minimizing desorption costs remains essential.

Moisture stability under realistic operating conditions is another critical concern. Although triazole-based MOFs generally show better hydrolytic stability compared to many carboxylate-based frameworks because of strong metal–triazolate coordination bonds, prolonged exposure to humid, multi-component flue gas can still lead to gradual performance decay. Water molecules may competitively occupy Lewis acidic metal sites and nitrogen-rich adsorption sites, thereby reducing the number of accessible high-energy adsorption centers for CO_2_ capture. In addition, acidic impurities such as SO_2_ and NO_x_ may bind more strongly than CO_2_ to exposed metal sites, potentially causing irreversible adsorption, pore blockage, or framework degradation during repeated adsorption–desorption cycles. Benchmark systems such as CALF-20 have demonstrated excellent tolerance to moderate humidity, but systematic long-term cyclic studies under realistic flue-gas compositions remain limited for most triazole-based MOFs. Future work should therefore prioritize breakthrough experiments, extended cycling tests, and multi-component adsorption studies under industrially relevant conditions to better assess long-term durability and practical viability.

From a synthetic perspective, most triazole-based MOFs are still produced by solvothermal methods that involve toxic solvents, long reaction times, and significant energy consumption. Although alternative routes such as mechanochemical and aqueous synthesis have been explored, challenges related to scalability, reproducibility, and precise structural control remain. Developing greener and more scalable synthesis strategies is therefore a key step toward practical deployment. In addition, the dynamic adsorption behavior of CO_2_ in flexible or ultramicroporous triazole-based MOFs is not yet fully understood. Phenomena such as gate-opening, cooperative adsorption, and framework flexibility require further investigation using advanced in situ characterization techniques and molecular simulations. Long-term structural integrity under repeated cycling also deserves greater attention, as many studies report recyclability over only a limited number of cycles.

Taken together, future research should focus on the rational design of triazole-based MOFs through integrated experimental and computational strategies, with particular emphasis on achieving an optimal balance between porosity, functionality, stability, and regenerability. Equally important is the development of scalable and environmentally friendly synthesis methods, together with systematic performance evaluation under realistic operating conditions, to facilitate the transition from laboratory-scale studies to industrial CO_2_ capture applications.

## 10. Conclusions

Triazole-based MOFs have established themselves as a versatile class of porous materials for CO_2_ capture, benefiting from the combination of nitrogen-rich coordination environments, tunable pore structures, and high chemical and thermal stability. The incorporation of triazole ligands introduces abundant nitrogen donor sites that contribute to CO_2_ adsorption through a range of interactions, including Lewis acid–base interactions, dipole–quadrupole interactions, and hydrogen bonding with framework components and functional groups. The adsorption performance of these materials is increasingly recognized as a result of the interplay between multiple structural parameters rather than a single descriptor such as surface area. Factors including metal identity, pore dimensions, confinement effects, linker functionality, and framework topology collectively determine CO_2_ affinity, selectivity, and regeneration behavior. In particular, the coexistence of OMSs and nitrogen-rich triazole sites can generate cooperative adsorption environments, providing stronger yet more selective interactions with CO_2_ molecules. Furthermore, strategies based on ultramicropore engineering and mixed-linker design have demonstrated that carefully controlled confinement and chemical heterogeneity can significantly improve adsorption performance. Despite these advances, several challenges remain for the practical deployment of triazole-based MOFs. Achieving an appropriate balance between adsorption strength and regeneration energy, maintaining structural integrity under humid and multicomponent gas conditions, and developing scalable and sustainable synthesis methods are among the key requirements for future development. In addition, a more comprehensive understanding of the relationship between framework structure, adsorption mechanisms, and long-term performance is needed to guide rational material design. In general, triazole-based MOFs provide a promising platform for the development of advanced CO_2_ capture materials by enabling precise control over both pore architecture and adsorption chemistry. Future progress will likely rely on the integration of computational screening, advanced in situ characterization methods, and environmentally responsible synthesis approaches to bridge the gap between fundamental studies and practical carbon capture technologies.

## Figures and Tables

**Table 1:** Comparative evaluation of synthetic methodologies for triazole-based MOFs considering structural control, reaction efficiency, environmental impact, and scale-up feasibility [41,42,43,44,45,46,47].

Synthetic Method	Typical Conditions	Crystallinity/Morphology	Reaction Time/Efficiency	Environmental Impact	Scale-Up and Industrial Feasibility
**Solvothermal/hydrothermal**	Typically carried out at 80–200 °C in sealed autoclaves using DMF, DMAc, DEF, alcohols, or water as the reaction medium.	Produces highly crystalline MOFs with good control over crystal size and framework structure. It is also the preferred method for obtaining single crystals suitable for structural analysis.	Reactions generally require several hours to a few days. Although product yields are often high, batch processing limits overall productivity.	Requires relatively large amounts of solvent, particularly organic solvents, which increases environmental concerns. Hydrothermal synthesis is a greener alternative but still involves considerable energy consumption.	This is the most established synthesis route and has been demonstrated from laboratory to pilot scale for several MOFs. Nevertheless, solvent recovery, energy demand, and the batch nature of the process remain major barriers to large-scale manufacturing.
**Microwave-assisted**	Conducted at approximately 100–200 °C using microwave dielectric heating in batch or continuous-flow systems.	Rapid and uniform heating promotes fast nucleation, often producing small particles with relatively narrow size distributions. Careful optimization is still required to avoid unwanted phases.	Reaction times are dramatically reduced, often to only a few minutes, resulting in higher productivity and improved space-time yields compared with conventional heating methods.	The shorter processing time lowers overall energy consumption and makes this approach more environmentally friendly than traditional solvothermal synthesis.	Continuous-flow microwave reactors have improved the prospects for large-scale production. However, equipment cost, temperature uniformity, and process control still present challenges for industrial implementation.
**Mechanochemical** (**LAG**)	Usually performed at room temperature by grinding or ball milling with a small amount of liquid additive, followed by optional aging if required.	Many MOFs can be obtained with good crystallinity, although particle morphology, phase purity, and the formation of complex structures are strongly influenced by milling conditions.	Synthesis is typically completed within minutes to a few hours, providing high product yields and excellent productivity because little or no solvent is required.	Considered one of the greenest MOF synthesis methods due to its minimal solvent use and relatively low energy requirements.	This approach shows strong potential for industrial production since milling, extrusion, and other continuous mechanical processes are already commercially available. Remaining challenges include ensuring consistent activation, maintaining phase purity, and translating laboratory protocols into reliable large-scale processes.

**Table 2:** Comparative CO_2_ capture performance of representative triazole-based MOFs and post-synthetically modified frameworks, highlighting structure–property relationships including pore confinement, metal centers, and functional group effects.

MOF/Material	Structural Feature	CO_2_ Uptake (mmol/g, Typical Conditions)	CO_2_/N_2_ Selectivity (IAST Unless Stated)	Q_st_ (kJ/mol)	Key Adsorption Mechanism	Ref.
**ZnATZoxH_2_O**	Zn–triazolate 2D layers + oxalate pillars, ultramicroporous channels	~2.76 (298 K, 1 bar)	>10^3^	44.5	Size exclusion + –NH_2_ + ultramicropore confinement	[67]
**Ni-Datz-MOF-74**	Ni-MOF-74 derivative with triazole functionalization	7.76 (1 bar)	327	34.7–49.7	OMS + triazole –NH_2_ synergy	[34]
**ZSTU-21** (**Cu-triazolate**)	Cu(II) PtS-type framework with dual channels	~2.76 (298 K, 1 bar)	105	28–31	Cu OMS + N-rich triazole interactions	[68]
**Cu/Zn–DATRZ–BTC MOF**	Mixed-linker hierarchical Cu/Zn MOF	4.06 (298 K, 1 bar)	20.6	15–23	OMS + amino-triazole + BTC synergy	[69]
**ZnF**(**TZ**)	Pure Zn–triazolate (rod-SBU)	0.27 (298 K; 0.15 bar)	13	23.6	Weak N sites, low polarity	[70]
**ZnF**(**aTZ**)	Amino-functionalized triazolate	0.56 (298 K; 0.15 bar)	46	32.3	–NH_2_ electrostatic + H-bonding	[70]
**ZnF**(**daTZ**)	Diamino-triazolate	0.96 (298 K; 0.15 bar)	120	33	High N density + confinement	[70]
**MAF-66**	3D dia triazolate framework	4.41 (298 K, 1 bar); 6.26 (273 K, 1 bar)	225 (298 K); 403 (273 K)	26–30	Dense N sites + strong confinement	[71]
**Zn_2_**(**atz**)**_2_**(**CO_3_**)	Carbonate-pillared Zn–triazolate layers	~1.9 (298 K, 15 kPa)	~188	~37.5	CO_3_^2−^ + –NH_2_ cooperative binding	[72]
**NICS-24**	Zn–triazolate + oxalate ultramicroporous framework	~3.0 (298 K, 1 bar)	~8	~68	Strong H-bonding + confinement	[73]
**ZnDatzBdc**	Flexible pillared-layer MOF	~2.05 (298 K)–2.89 (273 K)	107 (273 K); 129 (298 K)	26–29	Gate-opening + –NH_2_ interactions	[74]
**Zn_2_**(**C_2_O_4_**)(**triazolate**)**_2_**	Oxalate + triazolate 3D framework	~3.78 (293 K, 1.2 bar); ~4.35 (273 K, 1.2 bar)	Not reported	~40.8	Electrostatics + confinement	[75]
**CALF-20**	Zn–triazolate + oxalate ultramicroporous framework	~4.07 (293 K, 1.2 bar)	~230	~39	Dispersion + ultramicropore confinement	[76]
**SquCALF-20**	Squarate-modified CALF-20	~4.9 (293 K, 1 bar)	~500	~38	Enhanced polarity + tighter pores	[77]
**Zn–ABTC–Tz MOF**	Mixed triazole + carboxylate (anionic framework)	~2.07 (298 K, 1 bar); ~4.11 (273 K, 1 bar)	~5.1–5.5 (CH_4_ selectivity)	~25	Dual SBUs + electrostatics	[78]
**Triazole@UiO-67**	Post-synthetic triazole-functionalized Zr-MOF	~4.2 (298 K, 1 bar)	Not reported	~37.3	Electrostatics + confinement + polarity	[79]
**Benzotriazole@ZIF-8**	Post-synthetic linker exchange	1.77 (273 K, 1.2 bar)	Increased vs ZIF-8	10.47 (273 K); 13.70 (298 K)	N-rich pore functionalization	[80]
**MIL-68**(**In**)**-NHTr**	Triazole-functionalized MIL-68(In)	~3.7 (273 K, 1 bar)	Improved	~32.5	Lewis basic N sites + 1D channels	[81]

**Table 3:** Comparative CO_2_ adsorption performance of selected MOF families.

MOF Family	Representative Material(s)	CO_2_ Uptake (mmol/g)	CO_2_/N_2_ Selectivity	Q_st_ (kJ/mol)	Main Adsorption Feature	Advantages	Limitations	Refs.
**Triazole-based MOFs**	ZnATZoxH_2_O, Zn_2_(atz)_2_(CO_3_), ZnF(daTZ), CALF-20	~1–4	120–230	33–41	N-rich triazole sites + ultramicroporous confinement	High stability, strong low-pressure affinity, moderate regeneration energy	Functionalization may reduce porosity	[67,70,72,76]
**MOF-74 family**	Mg-MOF-74, Ni-MOF-74	3–5.3	32–182	38–47	OMSs	Very high uptake, strong CO_2_ binding	Moisture sensitivity, higher regeneration energy	[82,83]
**ZIF family**	ZIF-DIA, ZIF-L	0.9–6.2	7–20	6–23	Hydrophobic microporous framework	Exceptional stability, easy regeneration	Low low-pressure CO_2_ affinity	[84,85]
**UiO family**	UiO-66, NH_2_-UiO-66	1.1–3.3	37–120	24–28	Functionalized pore surfaces	Excellent chemical/hydrothermal stability	Moderate uptake and selectivity	[86,87]
**SIFSIX family**	SIFSIX-3-Cu	2.3–2.5	140–>1200	32–54	Strong electrostatic fluorinated channels	Ultra-high selectivity	High binding strength increases regeneration cost	[88,89,90]
**MIL family**	MIL-101, MIL-100	1.5–5.8	4–12	38–63	Large pore volume frameworks	High capacity	Limited selectivity	[91,92,93,94]

## Data Availability

No new data were created or analyzed in this study.
